# Costs of reproduction are present but latent in eusocial bumblebee queens

**DOI:** 10.1186/s12915-023-01648-5

**Published:** 2023-07-10

**Authors:** David H. Collins, David C. Prince, Jenny L. Donelan, Tracey Chapman, Andrew F. G. Bourke

**Affiliations:** School of Biological Sciences, University of East Anglia, Norwich Research Park, Norwich, NR4 7TJ UK

**Keywords:** Age-related gene expression, *Bombus terrestris*, Costs of reproduction, Eusociality, Evolutionary theory of ageing, mRNA-seq

## Abstract

**Background:**

The standard evolutionary theory of ageing proposes that ageing occurs because of a trade-off between reproduction and longevity. Eusocial insect queens exhibit positive fecundity-longevity associations and so have been suggested to be counter-examples through not expressing costs of reproduction and through remodelling conserved genetic and endocrine networks regulating ageing and reproduction. If so, eusocial evolution from solitary ancestors with negative fecundity-longevity associations must have involved a stage at which costs of reproduction were suppressed and fecundity and longevity became positively associated. Using the bumblebee (*Bombus terrestris*), we experimentally tested whether queens in annual eusocial insects at an intermediate level of eusocial complexity experience costs of reproduction, and, using mRNA-seq, the extent to which they exhibit a remodelling of relevant genetic and endocrine networks. Specifically, we tested whether costs of reproduction are present but latent, or whether a remodelling of relevant genetic and endocrine networks has already occurred allowing queens to reproduce without costs.

**Results:**

We experimentally increased queens’ costs of reproduction by removing their eggs, which caused queens to increase their egg-laying rate. Treatment queens had significantly reduced longevity relative to control queens whose egg-laying rate was not increased. Reduced longevity in treatment queens was not caused by increased worker-to-queen aggression or by increased overall activity in queens. In addition, treatment and control queens differed in age-related gene expression based on mRNA-seq in both their overall expression profiles and the expression of ageing-related genes. Remarkably, these differences appeared to occur principally with respect to relative age, not chronological age.

**Conclusions:**

This study represents the first simultaneously phenotypic and transcriptomic experimental test for a longevity cost of reproduction in eusocial insect queens. The results support the occurrence of costs of reproduction in annual eusocial insects of intermediate social complexity and suggest that reproductive costs are present but latent in queens of such species, i.e. that these queens exhibit condition-dependent positive fecundity-longevity associations. They also raise the possibility that a partial remodelling of genetic and endocrine networks underpinning ageing may have occurred in intermediately eusocial species such that, in unmanipulated conditions, age-related gene expression depends more on chronological than relative age.

**Supplementary Information:**

The online version contains supplementary material available at 10.1186/s12915-023-01648-5.

## Background

The standard evolutionary theory of ageing (ETA) proposes that ageing occurs because individuals have limited resources to invest in maximising both fecundity and longevity, selection prioritises reproductive success over survival, and the strength of selection against ageing weakens with age [1–5]. Along with declining performance and increasing mortality with age (ageing), an outcome of these factors is a fecundity-longevity trade-off. Specifically, reproduction imposes costs by reducing fecundity and/or longevity later in life, leading to a negative fecundity-longevity relationship [4].

The generality of this relationship has been challenged because, in theory, trade-offs between fecundity and longevity can become uncoupled and because, in some species, positive fecundity-longevity relationships occur [6–8]. For example, such relationships might occur within populations in which individuals vary in overall resource levels and well-resourced individuals invest in both high fecundity and high longevity. Nonetheless, in such populations a fecundity-longevity trade-off might still occur within individuals because no individual’s resources are limitless [9, 10].

Another important potential exception is represented by eusocial insects (those with a worker caste) such as eusocial Hymenoptera (ants, bees, and wasps) and termites. In these, reproductive phenotypes (queens or kings) are highly reproductive and comparatively long-lived, whereas workers are sterile or less reproductive and comparatively short-lived [11–14]. Furthermore, there appears to be a positive fecundity-longevity relationship within each caste, such that the most reproductive queens and workers (which in many eusocial Hymenoptera can reproduce asexually) live longer than the least reproductive queens and workers, respectively [15–26]. Therefore, it has been suggested that, in eusocial insects, queens (and reproductive workers) represent an exception to the usual negative fecundity-longevity relationship seen in other species, through not exhibiting costs of reproduction [22, 24, 27]. This is supported by studies in *Cardiocondyla* ants that showed that (a) queens experimentally induced to increase their costs of reproduction did not show decreased longevity [24] and (b) queens exhibit a peak in sexual production in late life [28]. Such phenomena do not appear to stem entirely from workers bearing the costs of reproduction for queens, because isolated reproductive *Cardiocondyla* queens showed greater longevity than isolated non-reproductive queens [21]. Moreover, even if workers absorb queens’ costs of reproduction, queens must still have evolved to reproduce without manifesting such costs personally.

The apparent abolition in eusocial insects of the conventional fecundity-longevity trade-off has therefore been hypothesised to result from a remodelling of the conserved genetic and endocrine networks that regulate ageing and reproduction [13, 29–34]. Recently, two such networks/gene sets have been proposed. One (the TOR/IIS-JH-Lifespan and Fecundity or TI-J-LiFe network) contains the nutrient-sensitive target of rapamycin (TOR) and insulin/insulin-like signalling (IIS) pathways, which, in solitary insects, affect (via Juvenile Hormone signalling) ageing and reproduction through activation of immune and antioxidant pathways [34, 35]. The other is a related enzymatic antioxidant gene set [36]. Both these sets of ageing-related genes (i.e. genes in pathways that directly affect ageing) include genes that have been found to be age-related (i.e. genes that change expression with age) in several eusocial insect species [34, 36].

If eusocial insects have indeed reversed the negative fecundity-longevity association found in solitary species via a remodelling of genetic and endocrine networks regulating ageing and reproduction, the question arises as to the stage in eusocial evolution at which this has occurred [37, 38]. Eusociality has evolved from non-social, typically annual life cycles, via ‘primitive’ eusociality with a low degree of reproductive division of labour between queen and workers, into ‘advanced’ eusociality with perennial life cycles and a high degree of queen-worker reproductive division of labour [39, 40]. Within this scheme, the annual eusocial bumblebees (*Bombus* spp.) appear to exhibit a degree of eusocial complexity intermediate between those of primitively eusocial species and advanced ones such as ants or the honeybee *Apis mellifera* [39, 41–43]. Therefore, bumblebee species represent highly suitable systems in which to investigate the evolution of ageing, longevity, and the fecundity-longevity trade-off over the course of eusocial evolution.

In the bumblebee *B. terrestris*, queens exhibit a positive relationship between lifetime reproductive success and longevity [16]. Moreover, a recent study aimed to test experimentally for costs of reproduction in reproductive *B. terrestris* workers. It found that, as in other species, workers in unmanipulated colonies exhibited a positive fecundity-longevity relationship, i.e. workers with more active ovaries lived longer [23]. However, this positive relationship was reversed (became negative) when randomly selected workers were experimentally manipulated to activate their ovaries. These results suggested that workers exhibit costs of reproduction but that workers choosing to reproduce in unmanipulated colonies are intrinsically high-quality individuals that can overcome such costs and achieve both high fecundity and high longevity. In other words, the results suggested that costs of reproduction might be present but unexpressed (latent) in such individuals, implying condition dependence of fecundity-longevity associations [23]. By extension, as queen-destined larvae receive nutrition of higher quality and/or in greater quantity [44], such that adult queens are likely to be intrinsically well-resourced, high-quality individuals, the results raised the possibility that queens in species such as *Bombus* also exhibit latent costs of reproduction [23].

In the current study, using *B. terrestris* as our model system, we therefore sought to test whether or not queens in eusocial insects at an intermediate level of eusocial complexity experience costs of reproduction, and the extent to which they exhibit a remodelling of genetic and endocrine networks regulating ageing and reproduction. Hence we conducted an experiment that manipulated queens’ costs of reproduction while profiling age-related gene expression changes in manipulated and control queens. Specifically, we aimed to discriminate between two alternative hypotheses. The first (H1) derives from ETA and states that a positive fecundity-longevity relationship in queens of species at the eusocial level of *B. terrestris* can arise because the costs of reproduction are present but latent. The second (H2) posits that queens in such species already represent a full exception to the trade-offs predicted by ETA because a remodelling of relevant genetic and endocrine networks has allowed them to reproduce without costs. Our study represents the first to test, simultaneously at phenotypic and transcriptomic levels, for a longevity cost of reproduction in eusocial insect queens.

**Fig. 1:**
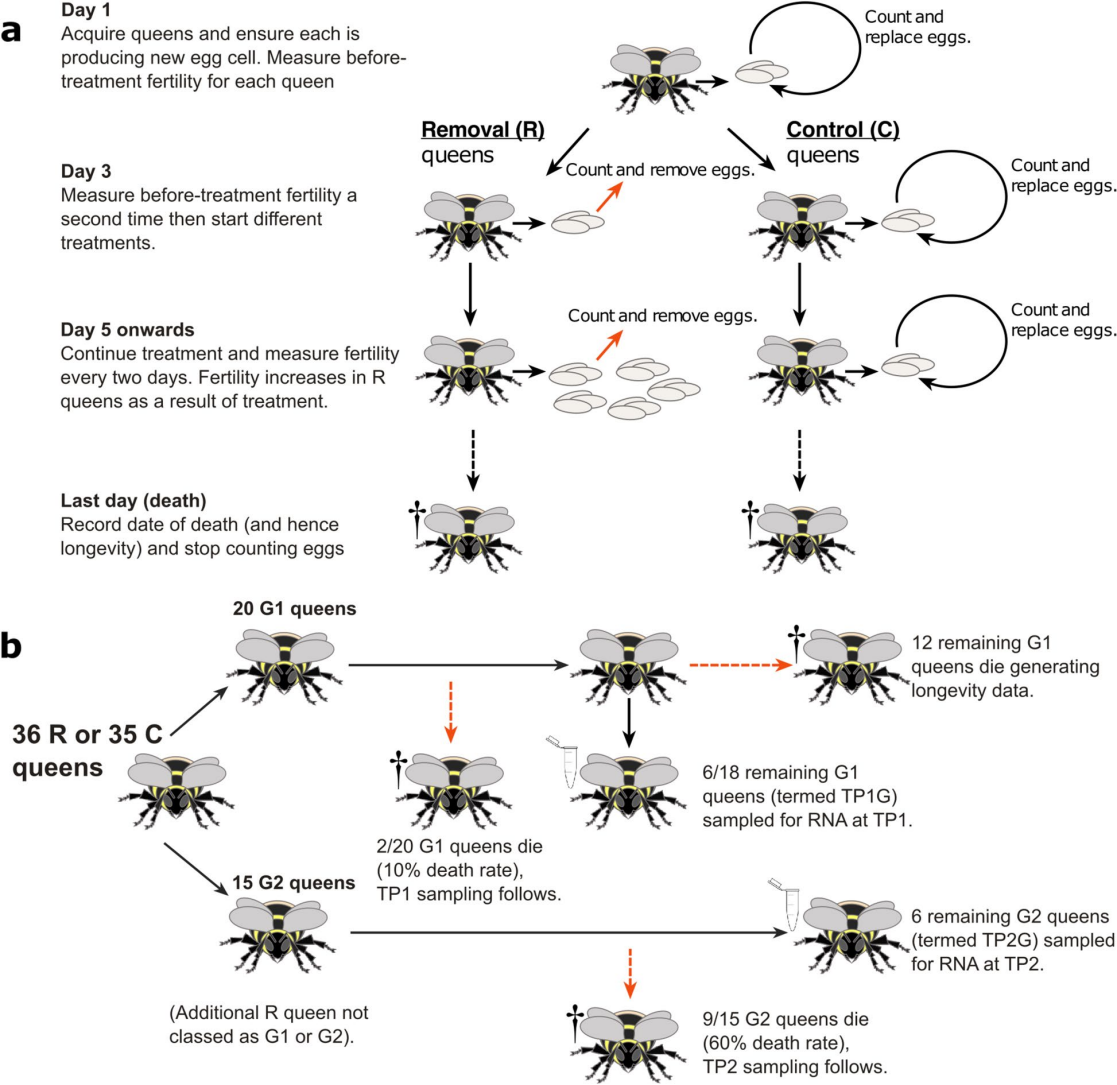
Outline of experimental design to test for costs of reproduction in *Bombus terrestris* queens. **a** Experimental treatments: R: Removal queens: eggs removed and counted; C: Control queens: eggs removed, counted, and replaced. Day 1 was the first day that new egg cells were observed in all colonies. **b** RNA sample collection strategy. G1, G2: random subgroups of queens within treatments. TP1, TP2: time-points for sampling of queens for RNA extraction (R queens: TP1 on day 37, TP2 on day 85; C queens: TP1 on day 89, TP2 on day 134). TP1G, TP2G: subsets of queens sampled for RNA extraction at TP1 and TP2, respectively. One R queen (Q1) was not assigned to G1 or G2, and was used to provide life-history data only. One C queen (Q57 in the G1 subgroup) was censored and therefore not included in longevity analyses or sampled for RNA. Final sample sizes were: R:TP1G (*N* = 6), R:TP2G (*N* = 6), R:life-history (*N* = 2 + 9 + 12 + 1 = 24); C:TP1G (*N* = 6), C:TP2G (*N* = 6), C:life-history (*N* = 2 + 9 + 12 − 1 = 22). See ‘Methods’ for full details

We manipulated queens’ costs of reproduction by experimentally removing eggs, which in *B. terrestris*, as in *C. obscurior* [24], has been shown to increase queens’ fertility (realised fecundity, as measured by egg-laying rate) [45]. We allocated queens to two treatments. In Removal (R) queens, all eggs were removed from the colony, therefore inducing queens to increase their fertility and so experience greater costs of reproduction (if present). In Control (C) queens, all eggs were removed from the colony and then replaced to control for disturbance (Fig. 1a). We then measured the queen’s longevity in each colony. To control for potential effects of egg removal on workers’ aggression to queens [45] and on queens’ overall activity levels, in both R and C colonies we also replaced egg-laying and aggressive workers with callow (newly eclosed) workers [46] and periodically measured queens’ locomotory activity and response to disturbance. To control for potential effects of colony size, we maintained the number of workers within each colony at 20 by removing any excess, newly eclosed workers and/or adding callow workers. To characterise changes in gene expression profiles with age brought about by the treatment, we sampled a subset of queens in both treatments at two time-points, the first when 10% queen mortality had occurred (time-point 1, TP1) and the second when 60% queen mortality had occurred (time-point 2, TP2) (Fig. 1b). From each queen, we extracted RNA from brain, fat body, and ovaries, since these tissues show gene expression changes with age in eusocial insects including *B. terrestris* [30, 34, 36, 47]. We then used mRNA-seq to characterise changes in the gene expression profiles over time in both R and C queens and to compare the resulting *B. terrestris* gene lists with age-related genes from other non-social and eusocial insects and ageing-related genes from the GenAge database [48], which catalogues experimentally validated, ageing-related genes in *Drosophila melanogaster*, and from the TI-J-LiFe network and enzymatic antioxidant gene set [34, 36].

H1 predicted that R queens should exhibit reduced longevity relative to C queens and dissimilar patterns of change in gene expression profile with age, especially for known ageing-related genes (as the egg removal treatment should cause R queens to express their latent costs of reproduction affecting longevity). By contrast, H2 predicted that R and C queens should exhibit equal longevities and that their patterns of change in gene expression profile with age should be similar, again especially for known ageing-related genes (as costs of reproduction affecting longevity, latent or otherwise, are absent). Even under H2, R queens would be expected to differ from C queens as regards expression differences in genes specifically associated with R queens’ increased egg-laying rate. Therefore, as well as testing for overall gene expression profile differences, we investigated differences between R and C queens in Gene Ontology of differentially expressed genes and tested whether patterns of change in gene expression profiles differed between R and C queens with respect to known ageing-related genes, i.e. those in the GenAge database, TI-J-LiFe network and enzymatic antioxidant gene set.

## Results

### Queen fertility and colony fertility

**Fig. 2:**
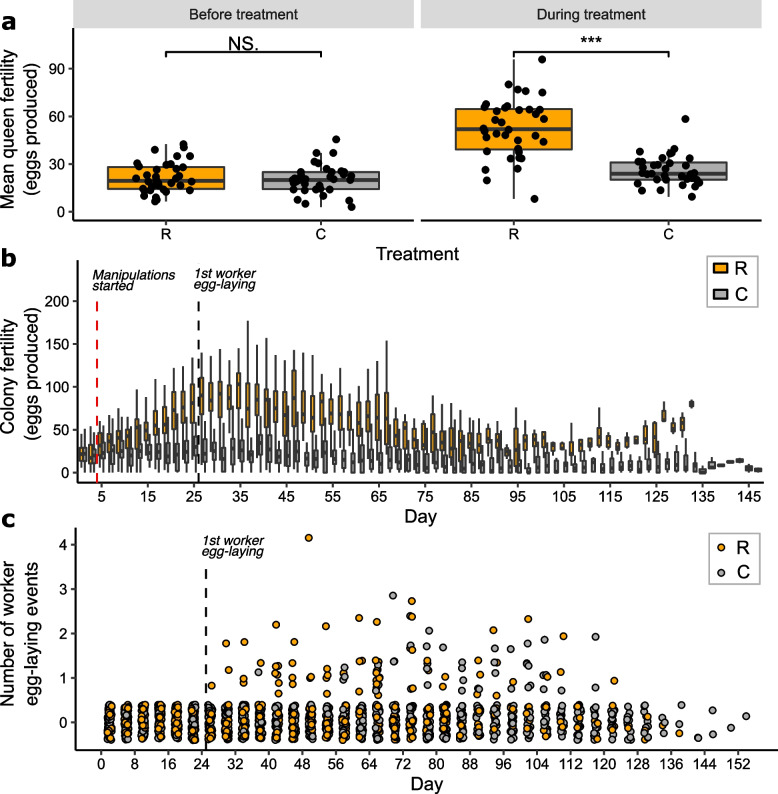
Fertility measures for R (eggs removed) and C (eggs removed and replaced) *Bombus terrestris* queens/colonies. **a** Queen fertility (mean number of eggs produced per 48-h period) for R (*N* = 36) and C (*N* = 35) queens before treatment started (baseline mean queen fertility: days 1 and 3) and during treatment until the first worker egg-laying was observed (during-treatment mean queen fertility: days 5–25 inclusive). Queen fertility over days 5–25 represented the fertility response of queens alone to the treatment. NS, not significant; *** *p* < 0.001. Black circles represent means for individual queens. **b** Colony fertility over time for R (*N* = 36 on day 1 declining to *N* = 1 on day 134) and C (*N* = 35 on day 1 declining to *N* = 1 on day 148) colonies until day 147. Red dashed line: day 5, manipulations started; black dashed line: day 26, when worker egg-laying first observed in any colony. Daily sample sizes are in Additional file 2: Table S23. Outliers not shown. For **a** and **b**, black horizontal bars: medians; boxes: interquartile ranges; whiskers: 1.5 × interquartile range. R colonies had significantly higher colony fertility than C colonies after treatment had started (see ‘Results’). **c** Observed worker egg-laying over time, recorded every 4 days during 10-min observations in R (*N* = 36 on day 1 declining to *N* = 1 on day 134) and C (*N* = 35 on day 1 declining to *N* = 1 on day 148) colonies. Black dashed line: day 26, when worker egg-laying first observed in any colony. Points are offset around each integral value on the *Y* axis. From day 26, R colonies had significantly more worker egg laying events than C colonies (see ‘Results’)

There was no significant difference in baseline mean queen fertility (queen egg production before the treatment started) between R and C colonies (mean [95% CI] eggs per 48-h period: R, 21.5 [18.8, 24.7]; C, 20.3 [17.7, 23.4]; negative binomial glmm: *b* = 0.057, Seb = 0.099, *z* = 0.578, *p* = 0.563; Fig. 2a). However, there was a significant increase in during-treatment mean queen fertility (queen egg production over days 5-25 inclusive) across both groups (*b* = 0.893, Seb = 0.094, *z* = 9.485, *p* < 0.001). This increase had a significant interaction with treatment (*b* = 0.650, Seb = 0.14, *z* = 4.766, *p* < 0.001), with R queens producing approximately twice as many eggs over days 5–25 as C queens (mean [95% CI] eggs per 48-h period: R, 52.5 [46.3, 59.6]; C, 25.9 [22.6, 29.7]) (Fig. 2a, Additional file 1: Fig. S1). This result showed that, as intended, the R treatment significantly increased queen egg-laying rate.

R colonies also had significantly higher colony fertility (overall colony egg production over the whole experimental period, including any worker-laid eggs) than C colonies (negative binomial glmm: *b* = 1.147, Seb = 0.078, *z* = 14.679, *p* < 0.001; Fig. 2b; Additional file 1: Figs. S2, S3; Additional file 2: Table S1). Worker egg-laying was first observed in any colony (out of all R and C colonies) on day 26, and, over the whole experimental period, was observed in 24/36 R colonies and 25/35 C colonies. We therefore (conservatively) took day 26 as the date beyond which, in all colonies, some differences in treatment-specific colony fertility could have been due to worker egg-laying (Fig. 2c, Additional file 1: Fig. S4). Consistent with this, after day 26, compared to C colonies, R colonies had significantly higher levels of observed worker egg-laying (zero-inflated negative binomial glmm: Treatment: *b* = 2.917, Seb = 0.831, *z* = 3.509, *p* < 0.001; Fig. 2c), significantly earlier onset of worker egg-laying (median onset of worker egg-laying in days [from day 1 in the experiment]: R, 44; C, 78; Cox’s proportional hazards analysis: hazards ratio = 13.32, *z* = 5.661, *p* < 0.001; Additional file 1: Fig. S5), and significantly higher numbers of egg-laying workers removed (mean [SD] workers per colony: R, 2.3 [2.5]; C, 1.5 [1.2]; negative binomial glmm: *b* =  − 0.991, Seb = 0.219, *z* =  − 4.531, *p* < 0.001). However, observed worker egg-laying was significantly time-dependent, peaking at day 76 and then declining for the rest of the experiment (Time: *b* = 40.156, Seb = 9.133, *z* = 4.397, *p* < 0.001; Fig. 2c). As R queens showed significantly higher fertility than C queens before day 26, and as colony fertility peaked on day 35 (Fig. 2b) whereas observed worker egg-laying did not peak until day 76, these results show that the R treatment continued to increase queen egg-laying rate after the start of worker egg-laying.

### Queen longevity, worker aggression, worker additions and removals, and queen activity/response to disturbance

**Fig. 3:**
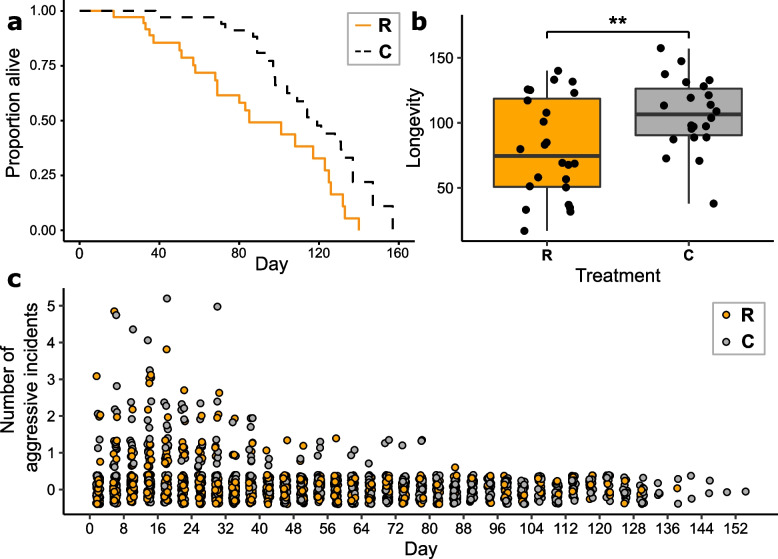
Queen longevity and worker aggression for experimental *Bombus terrestris* queens/colonies. **a** Survivorship of R (eggs removed; *N* = 24) and C (eggs removed and replaced; *N* = 22) queens; **b** Longevity (days from start of experiment) of R (eggs removed; *N* = 24) and C (eggs removed and replaced; *N* = 22) queens. Black horizontal bars: medians; boxes: interquartile ranges; whiskers: 1.5 × interquartile range. ** *p* < 0.01. In **a** and **b**, R queens had significantly reduced survivorship/longevity relative to C queens (see ‘Results’); **c** Observed worker-to-queen aggression over time (recorded every 4 days during 10-min observations) in R (eggs removed; *N* = 36 on day 1 and *N* = 1 on day 134) and C (eggs removed and replaced; *N* = 35 on day 1 and *N* = 1 on day 148) colonies. Daily sample sizes are in Additional file 2: Table S23. Points are offset around each integral value on the *Y* axis. There was no significant difference in observed worker-to-queen aggression between R and C colonies (see ‘Results’)

R queens had significantly reduced longevity relative to C queens (median longevity [from day 1 of the experiment]: R, 73.6 days; C, 105.4 days; Cox’s proportional hazards analysis: hazards ratio = 0.224, *z* =  − 4.287, *p* < 0.001; Fig. 3a, b). There was no significant difference in observed worker aggression (zero-inflated negative binomial glmm: *b* = 0.056, Seb = 0.307, *z* = 0.184, *p* = 0.854; Fig. 3c), filmed worker aggression (binomial glmm: *b* = 0.453, Seb = 0.912, *z* = 0.497, *p* = 0.619, Additional file 1: Fig. S6), onset of worker aggression (Cox’s proportional hazards analysis: hazards ratio = 0.844, *z* =  − 0.649, *p* = 0.517; Additional file 1: Fig. S7), or number of workers removed due to aggression (mean [SD] workers per colony: R, 1.6 [1.7]; C, 2.1 [2.6]; negative binomial glmm: *b* =  − 0.135, Seb = 0.291, *z* =  − 0.462, *p* = 0.644) between R and C colonies. In several colonies (8/36 R colonies and 4/35 C colonies), the queen died before any worker aggression was observed. In addition, observed worker aggression was significantly time-dependent for both treatments (zero-inflated negative binomial glmm: *b* =  − 0.057, Seb = 0.001, *z* =  − 5.782, *p* < 0.001), peaking at day 18 before declining throughout the rest of the experiment (Fig. 3c), whereas mean queen longevity across both treatments was 89.4 days. There was also no significant difference in observed queen activity (binomial glmm: *b* =  − 0.163, Seb = 0.143, *z* =  − 1.143, *p* = 0.253; Additional file 1: Fig. S8), filmed queen activity (binomial glmm: *b* =  − 0.067, Seb = 0.297, *z* = 0.224, *p* = 0.822; Additional file 1: Fig. S9), or queens’ response to disturbance (binomial glmm: *b* =  − 3.3, Seb = 1.97, *z* =  − 1.678, *p* = 0.093; Additional file 1: Fig. S10) between R and C queens. Therefore, the reduction in queen longevity in the R treatment was not caused by greater worker aggression to queens or by differences in queen activity levels or responsiveness between treatments.

There were significantly fewer excess workers removed from R than from C colonies (mean [SD] workers per colony: R, 39.1 [19.1]; C, 67.9 [30.7]; negative binomial glmm: *b* = 0.373, Seb = 0.126, *z* = 2.971, *p* = 0.003). However, there was no significant difference between treatments in the numbers of either dead workers removed (mean [SD] workers per colony: R, 8.5 [7.8]; C, 11.5 [8.4]; negative binomial glmm: *b* =  − 0.146, Seb = 0.187, *z* =  − 0.780, *p* = 0.435) or callow workers added as replacements for egg-laying, aggressive, or dead workers (mean [SD] workers per colony: R, 8.8 [7.1]; C, 11.3 [8.2]; negative binomial glmm: *b* =  − 0.226, Seb = 0.200, *z* =  − 1.128, *p* = 0.259). In addition to the independent effect of treatment on queen longevity, excess workers removed was found to be significantly associated with queen longevity, with a higher number of excess workers removed being associated with reduced queen longevity (Cox’s proportional hazards analysis: hazards ratio = 1.797, *z* = 4.903, *p* < 0.001). As fewer excess workers were removed from R colonies than from C colonies, and the numbers of workers removed due to either egg-laying or aggression were low compared to the number of excess workers removed, and R and C colonies did not differ in numbers of dead workers removed or callow workers added, the reduction in longevity of R queens was not caused by adding workers to, or removing them from, experimental colonies.

Overall, these results support the prediction of H1 that queens in the R treatment, which experienced a greater cost of reproduction (from their higher egg-laying rates), should exhibit reduced longevity.

### Age-related gene expression

Across the 70 libraries created, mRNA-seq resulted in a mean of 55,280,953 read pairs per library for brain, 61,864,754 read pairs per library for fat body, and 69,025,352 reads pairs per library for ovaries (Additional file 2: Table S2). The libraries pseudoaligned to the *B. terrestris* transcriptome with a mean percentage pseudoalignment of 77.3% (range 49.4–88.4%) for brain, 58.7% (5.5–90.0%) for fat body (including the two fat body libraries that were excluded from further analysis), and 75.9% (32.7–83.1%) for ovaries (Additional file 2: Table S3).

**Fig. 4:**
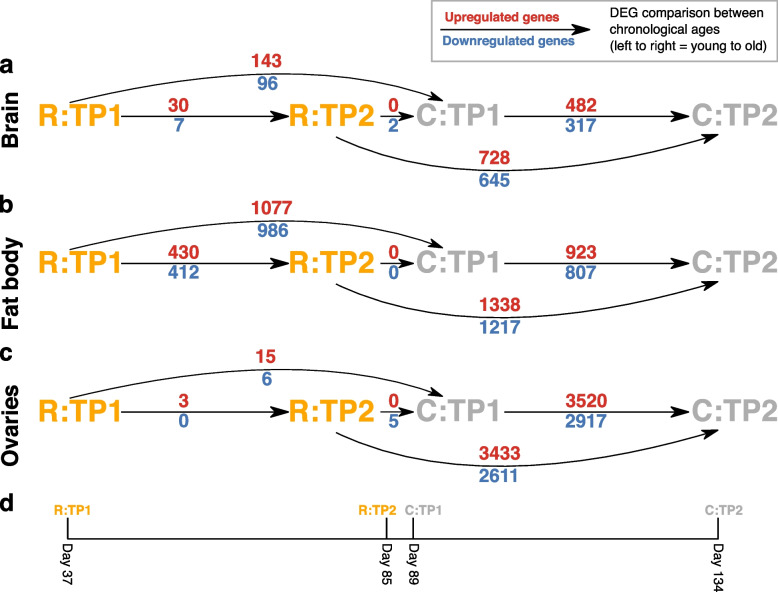
Comparisons of mRNA-seq gene expression profiles by chronological age in experimental *Bombus terrestris* queens. Arrows and associated numbers denote differentially expressed genes (DEGs) between two conditions (increasing in chronological age from left to right) for **a** brain; **b** fat body; and **c** ovaries. R conditions, eggs removed; C conditions, eggs removed and replaced. Red numbers, above arrows: numbers of genes upregulated (more expressed with chronological age) between two conditions linked by an arrow; blue numbers, below arrows: numbers of genes downregulated (less expressed with chronological age) between two conditions linked by an arrow. **d** Scale displaying the day of the experiment on which each condition was sampled. TP1: time-point 1, TP2: time-point 2. Sample sizes: brain: R:TP1G (*N* = 6), R:TP2G (*N* = 5); C:TP1G (*N* = 6), C:TP2G (*N* = 5); fat body: R:TP1G (*N* = 6), R:TP2G (*N* = 6); C:TP1G (*N* = 6), C:TP2G (*N* = 4); ovaries: R:TP1G (*N* = 6), R:TP2G (*N* = 6); C:TP1G (*N* = 6), C:TP2G (*N* = 6)

In total, between the two time-points (TP1 and TP2), and pooling across both treatments (R and C), there were 836 differentially expressed genes (DEGs) in brain, 2572 DEGs in fat body, and 6440 DEGs in ovaries. (All DEGs were determined using an FDR adjusted *p*-value threshold of 0.05.) In brain, in R queens: 30 genes were more expressed in TP2G than TP1G (i.e. genes which increased in expression with age; henceforth, ‘upregulated genes’) and 7 genes were more expressed in TP1G than TP2G (i.e. genes which decreased in expression with age; henceforth, ‘downregulated genes’); in C queens: 482 genes were upregulated and 317 genes were downregulated (Fig. 4a; Additional file 1: Fig. S11; Additional file 2: Table S4). In fat body, in R queens: 430 genes were upregulated and 412 genes were downregulated; in C queens: 923 genes were upregulated and 807 genes were downregulated (Fig. 4b; Additional file 1: Fig. S12; Additional file 2: Table S5). In ovaries, in R queens: 3 genes were upregulated and 0 genes were downregulated; in C queens: 3520 genes were upregulated and 2917 genes were downregulated (Fig. 4c; Additional file 1: Fig. S13; Additional file 2: Table S6).

**Fig. 5:**
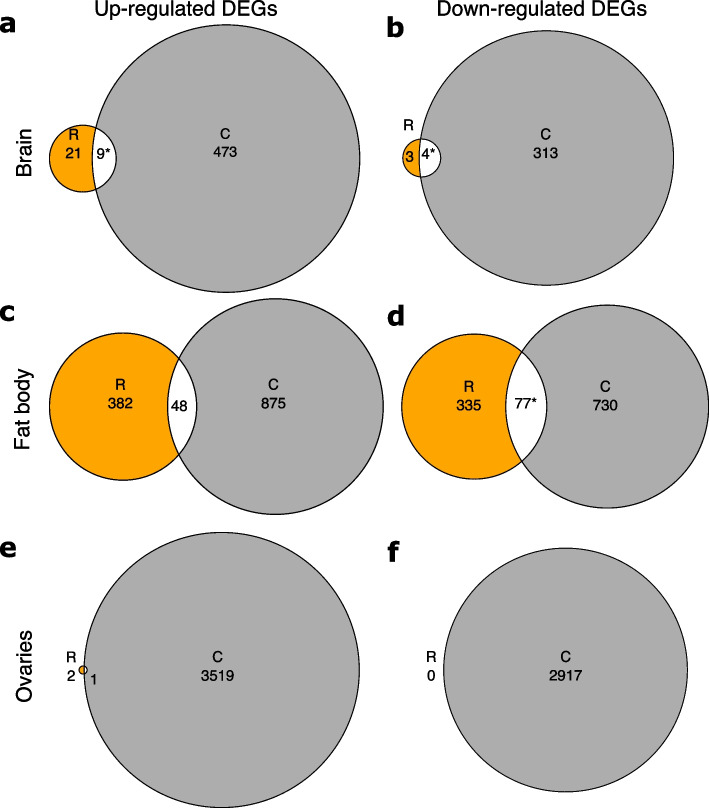
Comparison of changes in mRNA-seq gene expression profiles with relative age in *Bombus terrestris* queens. Euler diagrams of overlaps between differentially expressed genes (DEGs), i.e. genes differentially expressed between the two time-points TP1 and TP2 within treatments and shared between R queens (eggs removed) and C queens (eggs removed and replaced) for: **a**, **b** brain; **c**, **d** fat body; and **e**, **f** ovaries. (In panel **e**, because their low values mean there is a lack of space, numbers of DEGs for R queens and shared between R and C queens are shown adjacent to the relevant area.) Asterisks (*), significant overlap in DEGs (Fisher’s exact test, *p* < 0.05 after Bonferroni correction). Upregulated DEGs: DEGs significantly more expressed in TP2G than TP1G, i.e. that increase expression with queen relative age; downregulated DEGs: DEGs significantly more expressed in TP1G than TP2G, i.e. that decrease expression with queen relative age. Results of statistical tests are in Additional file 2: Table S7 and identities of overlapping genes are in Additional file 2: Table S8. Sample sizes: brain: R:TP1G (*N* = 6), R:TP2G (*N* = 5); C:TP1G (*N* = 6), C:TP2G (*N* = 5); fat body: R:TP1G (*N* = 6), R:TP2G (*N* = 6); C:TP1G (*N* = 6), C:TP2G (*N* = 4); ovaries: R:TP1G (*N* = 6), R:TP2G (*N* = 6); C:TP1G (*N* = 6), C:TP2G (*N* = 6)

**Fig. 6:**
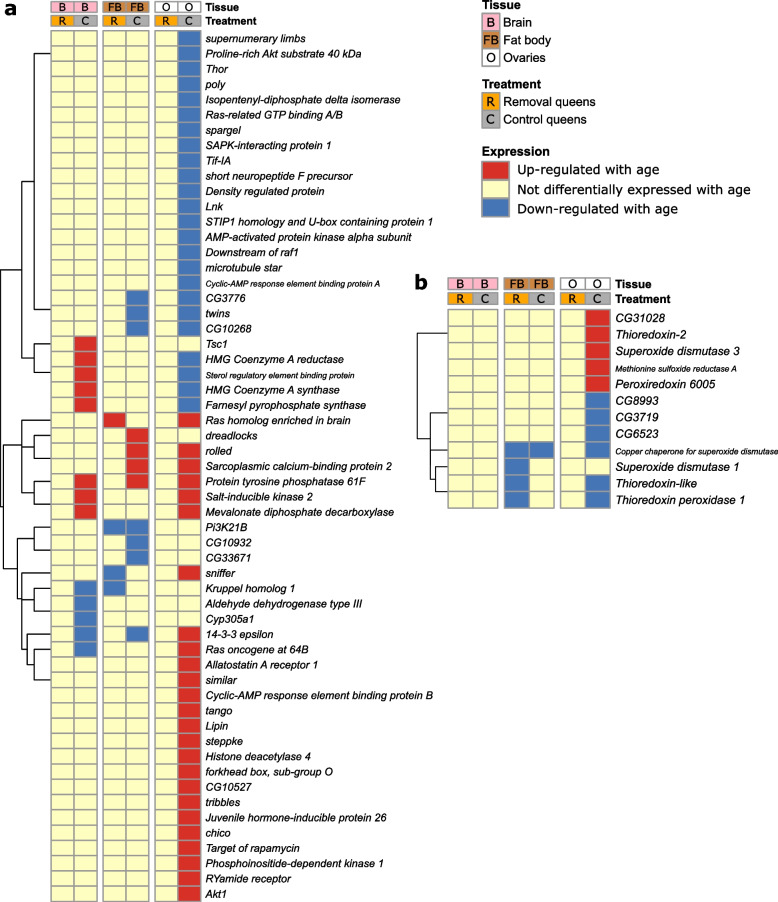
Age-related gene expression patterns compared between *Bombus terrestris* queens (current study) and ageing-related genes. **a** Comparison with TI-J-LiFe network; each row represents an individual gene from *Drosophila melanogaster* included in the TI-J-LiFe network by Korb et al. [34] that has a single-copy orthologue in *B. terrestris*; **b** comparison with enzymatic antioxidant gene set; each row represents an individual gene from *D. melanogaster* included in the enzymatic antioxidant gene set by Kramer et al. [36] that has a single-copy orthologue in *B. terrestris*. In **a** and **b**, each column shows the age-related expression status of the focal genes in a given treatment and tissue in the experimental *B. terrestris* queens. Vertical breaks separate the three tissues (brain, fat body and ovaries). The dendogram at left groups genes that cluster according to their gene expression patterns. Sample sizes: brain: R:TP1G (*N* = 6), R:TP2G (*N* = 5); C:TP1G (*N* = 6), C:TP2G (*N* = 5); fat body: R:TP1G (*N* = 6), R:TP2G (*N* = 6); C:TP1G (*N* = 6), C:TP2G (*N* = 4); ovaries: R:TP1G (*N* = 6), R:TP2G (*N* = 6); C:TP1G (*N* = 6), C:TP2G (*N* = 6)

Based on these findings, the mRNA-seq gene expression profiles showed that, in each tissue (brain, fat body, ovaries), R queens exhibited far less differential gene expression between TP1 and TP2 than C queens (Figs. 5 and 6; Additional file 1: Figs. S11 – S13). To determine more exactly whether gene expression profile change with age (from TP1 to TP2) differed between R and C queens, we compared lists of DEGs across R and C treatments within each tissue (Additional file 2: Tables S4 – S6). Overall, 3/6 comparisons of R and C DEGs showed no significant overlap and 3/6 showed significant overlap (Fig. 5; Additional file 2: Tables S7, S8). The significant overlaps were as follows: (a) in brain, 9/30 of DEGs upregulated in R were also upregulated in C (Fisher’s exact test, *α* = 0.0167, *p* = 5.33 × 10^−6^; Fig. 5a; Additional file 2: Table S7); (b) in brain, 4/7 of DEGs downregulated in R were also downregulated in C (Fisher’s exact test, *α* = 0.0167, *p* = 2.72 × 10^−5^; Fig. 5b; Additional file 2: Table S7); and (c) in fat body, 77/412 of DEGs downregulated in R were also downregulated in C (Fisher’s exact test, *α* = 0.0167, *p* = 2.16 × 10^−13^; Fig. 5d; Additional file 2: Table S7). However, levels of overlap were relatively low and there were at least double the number of DEGs (up- or downregulated) in C compared to R queens in each tissue (Fig. 5). Hence, consistent with H1, although some overlap occurred, R and C queens exhibited dissimilar patterns of change in their gene expression profiles with relative age (age measured in terms of percentage mortality). In this respect, the most informative tissues were brain and fat body, as, unlike ovaries, these tissues showed moderate to high age-related gene expression changes in both R and C queens, while still showing dissimilar patterns of change for each treatment (Fig. 5).

### Gene Ontology

To determine whether the biological functions of DEGs differed between treatments, we used Gene Ontology (GO) analysis to identify GO terms. Using OrthoFinder, we identified 6074 single-copy orthologues between *B. terrestris* and *D. melanogaster* (57.4% of the 10,591 genes expressed in the *B. terrestris* mRNA-seq libraries). We used these to isolate 248 non-redundant enriched GO terms for the DEGs (Additional file 2: Table S9).

Within each tissue where the comparison could be made, no enriched GO terms were shared between R and C queens (Additional file 2: Table S9). In brain, upregulated DEGs in R queens were not enriched for GO terms, while upregulated DEGs in C queens were enriched for GO terms in cytoplasmic translation (GO:0002181). Downregulated DEGs in R queens were enriched for GO terms associated with ‘ion transport’ (4/9 terms) and ‘amino acid processes’ (3/9 terms), while downregulated DEGs in C queens were not enriched for GO terms (Additional file 2: Table S9).

In fat body, upregulated DEGs in R queens were enriched for two GO terms: DNA recombination (GO:0006310) and cellular response to DNA damage stimulus (GO:0006974), while upregulated DEGs in C queens were enriched for a variety of processes with no obvious similarities between them. Downregulated DEGs in R queens were enriched for GO terms associated with organelle-related processes (10/15 terms), while downregulated DEGs in C queens were enriched for GO terms associated with ‘metabolic’ and ‘biosynthetic’ processes (7/10 terms) (Additional file 2: Table S9).

In ovaries, in R queens, GO analysis was not conducted as up- and downregulated genes comprised 3 and 0 DEGs, respectively. Upregulated DEGs in C queens were enriched for GO terms associated with ‘development’ (19/97 terms). Downregulated DEGs in C queens were enriched for GO terms associated with regulation of the ‘cell cycle’ including ‘nuclear’ and ‘chromosomal’ changes (18/45 terms) (Additional file 2: Table S9).

Across all GO enrichment analyses, only 3 of the 248 non-redundant GO terms were specifically related to reproduction. These were as follows: growth of a germarium-derived egg chamber (GO:0007295; in downregulated DEGs in R queen brain); embryo development ending in birth or egg hatching (GO:0009792; in upregulated DEGs in C queen ovaries); and eggshell chorion gene amplification (GO:0007307; in downregulated DEGs in C queen ovaries) (Additional file 2: Table S9). Therefore, reproduction-related GO terms were not specifically enriched in R queens, suggesting that differences in DEGs between R and C queens were not driven only by the greater egg-laying rate of R queens. Overall, consistent with H1, the biological functions of age-related DEGs (in brain and fat body), as reflected by dissimilarities in the associated GO terms, differed between R and C queens.

### Gene expression analysis: comparisons with age- and ageing-related genes from other species

To compare our data with those from previous studies, we tested whether *B. terrestris* R and C queens differed from one another as regards their expression patterns for age-related genes identified in previous studies of solitary (*D. melanogaster*) and eusocial (*A. mellifera*) insect species. For *D. melanogaster*, no comparisons in either R or C queens showed significant overlap between orthologues of DEGs and comparable tissue-specific mRNA-seq *D. melanogaster* studies [49, 50] (0/8 comparisons; Additional file 2: Tables S10, S11). Therefore, *B. terrestris* queens (across both treatments) and *D. melanogaster* females did not exhibit a shared set of age-related genes. For the orthologue of the *A. mellifera* age-related gene *vitellogenin*, R and C queens differed in that R queens showed no age-related differential expression in any tissue, whereas C queens showed no age-related differential expression in fat body and ovaries but showed downregulation with age in brain, which is in the direction opposite to that shown by *A. mellifera* queens. Hence, for this specific gene, R and C queens differed in age-related gene expression.

To further discriminate between H1 and H2 from the gene expression data, and to investigate potential remodelling of genetic networks regulating ageing, we tested whether patterns of change in gene expression profiles differed between R and C queens with respect to known ageing-related genes. In comparisons with genes from the *D. melanogaster* GenAge database [48], no comparisons of orthologues of DEGs in either R or C queens showed significant overlap (0/6 comparisons; Additional file 1: Fig. S14; Additional file 2: Tables S12, S13), rendering the comparisons uninformative as regards discriminating H1 and H2. In comparisons of DEGs with the TI-J-LiFe network [34], R queens did not show significant overlap in any of the three tissues, whereas C queens showed significant overlaps in brain (for 5/6 comparisons) but not fat body or ovaries (Fig. 6a; Additional file 2: Tables S14, S15). For specific TI-J-LiFe network genes that changed expression with age in at least one tissue in either R or C queens (57 genes), patterns of age-related gene expression also differed. In brain, in R, 0 genes were upregulated or downregulated; in C, 8 DEGs were upregulated and 5 DEGs were downregulated (Fig. 6a). In fat body, in R, 1 DEG was upregulated and 3 DEGs were downregulated; in C, 4 DEGs were upregulated and 7 DEGs were downregulated (Fig. 6a). In ovaries, in R, 0 genes were upregulated or downregulated; in C, 25 DEGs were upregulated and 24 DEGs were downregulated (Fig. 6a). For the enzymatic antioxidant gene set [36], R queens showed significant overlap in fat body (for one comparison, i.e. when the top 300 DEGs from each list were compared) but not in brain or ovaries, whereas C queens showed no significant overlaps in any of the three tissues (Fig. 6b; Additional file 2: Tables S16, S17).

Overall, therefore, in comparisons of DEGs in each queen treatment to ageing-related genes from the TI-J-LiFe network and enzymatic antioxidant gene set, R and C queens exhibited, consistent with H1, dissimilar patterns of change in their gene expression profiles with relative age. Specifically, DEGs in R queens, in contrast to those in C queens, showed no significant overlap with the TI-J-LiFe network (Fig. 6a). In addition, for DEGs in either R or C queens that did overlap with TI-J-LiFe network genes, R and C queens showed dissimilar patterns of gene expression change with age (i.e. R queens showed few changes and C queens showed many changes; Fig. 6a). For the comparison with the enzymatic antioxidant gene set, DEGs in R queens showed significant overlap but those in C queens did not (Fig. 6b).

### Gene expression analysis: relative versus chronological age

To investigate whether the age-related gene expression patterns observed between R and C queens were affected by sampling at time-points reflecting relative rather than chronological age (absolute age, measured in days from day 1) (Fig. 1), we determined the number of genes that were significantly differentially expressed between R:TP1 (day 37) and C:TP1 (day 89), between R:TP2 (day 85) and C:TP1 (day 89), and between R:TP2 (day 85) and C:TP2 (day 134) in each tissue (Fig. 4; Additional file 1: Fig. S15; Additional file 2: Tables S18—S20). Across the three tissues, between R:TP1 and C:TP1, there were moderate numbers of DEGs (15–1077 upregulated, 6–986 downregulated); between R:TP2 and C:TP1, there were few DEGs (0 upregulated, 2–5 downregulated); and between R:TP2 and C:TP2, there were large numbers of DEGs (728–3433 upregulated, 645–2611 downregulated) (Fig. 4; Additional file 1: Fig. S15; Additional file 2: Tables S18—S20). In addition, there were significant and high percentage overlaps in DEGs for which the time period (the span between two time-points) also overlapped (i.e. R:TP1 to C:TP1 versus R:TP1 to R:TP2; ranges across the three tissues of 66.7–89.5% for upregulated genes, 42.9–85.7% for downregulated genes; and R:TP2 to C:TP2 versus C:TP1 to C:TP2, ranges across the three tissues of 75.1–90.6% for upregulated genes, 75.4–83.3% for downregulated genes; Additional file 2: Tables S21, S22). As the gene expression differences between R:TP2 and C:TP1 were overall much smaller than for the other comparisons, and as queens sampled at these time-points had similar chronological ages but different treatments and relative ages (R:TP2: 85 days, 60% mortality; C:TP1: 89 days, 10% mortality), these and the other results (Fig. 5) imply that age-related changes in gene expression profile that were caused by the experimental treatment occurred predominantly with respect to queens’ relative age and not their chronological age. In turn, this suggests that age-related gene expression in *B. terrestris* queens typically shows a degree of invariance (i.e. relatively fixed pattern of change) with chronological age. However, although R:TP2 and C:TP1 exhibited similar expression profiles and overlapping time periods showed high percentage overlaps in their DEGs, the results were inconsistent with there being a common gene expression trajectory for all queens irrespective of treatment. This can be concluded because, in brain and fat body (though not ovaries), there were many more DEGs isolated between R:TP1 and C:TP1 than between R:TP1 and R:TP2, and there were many more DEGs isolated between R:TP2 and C:TP2 than between C:TP1 and C:TP2 (Fig. 4; Additional file 1: Fig. S15).

## Discussion

We experimentally manipulated queens of the intermediately eusocial bumblebee *B. terrestris* by removing their eggs, causing them to double their egg-laying rate (Fig. 2a). This increase in the costs of reproduction led to a significant decrease in queen longevity, which fell by 30% relative to that of control queens (to 73.6 days from 105.4 days, as measured from the experiment’s start day) (Fig. 3a, b). The decreased longevity of treatment queens was not caused by differences in worker aggression, queen activity, or queen responsiveness. Our results therefore support the hypothesis (H1) that a positive fecundity-longevity relationship in eusocial insect queens can arise because costs of reproduction are present but latent, with the high individual quality inherent in the queen phenotype allowing queens in normal conditions to overcome such costs and exhibit both high fecundity and high longevity. This explanation is consistent with ETA (see ‘Background’), and our results therefore imply that eusocial insects at stages of eusocial complexity short of advanced eusociality are not an exception to the predicted fecundity-longevity trade-off found in most non-social species. In addition, treatment queens differed from control queens in patterns of change in their gene expression profile with relative age by exhibiting: (i) dissimilar overall profile changes (Fig. 5); (ii) unalike GO terms not linked to reproduction; (iii) different patterns of expression change in *vitellogenin*; (iv) differential overlaps with ageing-related genes from the TI-J-LiFe network as well as different age-related gene expression patterns of TI-J-LiFe genes (Fig. 6a); and (v) differential overlaps with ageing-related genes from the enzymatic antioxidant gene set (Fig. 6b). These results support H1’s prediction that treatment and control queens should exhibit dissimilar patterns of change in gene expression profile with age, especially for known ageing-related genes. However, the results suggesting that, in unmanipulated queens, age-related changes in gene expression exhibit a degree of invariance with chronological age (Fig. 4; Additional file 1: Fig. S15) also raise the possibility, consistent with H2, that some remodelling of genetic and endocrine networks underpinning ageing has occurred in *B. terrestris*.

The occurrence of a longevity cost of reproduction in *B. terrestris* queens contrasts with the findings of Schrempf et al. [24] in *C. obscurior* ant queens, which showed no effect of increased costs of reproduction on longevity. As hypothesised (see ‘Background’), this difference is consistent with *B. terrestris* representing an intermediate stage of eusocial complexity, in contrast to the advanced eusociality found in ants. In other words, our results suggest that, in the change from a negative fecundity-longevity association as typically found in solitary insects to the positive one apparent in advanced eusocial insects, *B. terrestris* represents a stage at which (a) a condition-dependent positive fecundity-longevity association exists in queens (i.e. costs of reproduction remain present but are latent) and (b), conceivably, remodelling of the genetic and endocrine networks that regulate ageing and reproduction has occurred to the extent that age-related changes in gene expression profiles have become relatively invariant with chronological age, as we further discuss below.

In the current study, R colonies had significantly more egg-laying workers removed than C colonies (due to a higher rate of observed worker egg-laying), and significantly fewer excess workers removed (due to R colonies, given their eggs were removed, producing fewer workers), which may have affected the within-colony worker age-structure within each treatment and so have influenced the results. However, colony age-structure and/or worker removal is unlikely to have accounted for the difference in longevity between R and C queens for the following reasons. First, the number of egg-laying workers removed was low (means of 2.3 and 1.5 workers removed, respectively, from R and C colonies) and therefore unlikely to have substantially altered worker age-structure given that each colony was maintained at a size of 20 workers. Accordingly, the number of egg-laying workers removed was not significantly associated with queen longevity. Second, although there were significantly fewer excess workers removed from R colonies than C colonies (means of 39.1 and 67.9 workers removed, respectively), all the removed workers were 0–2 days old, and their removal was therefore unlikely to have affected worker age-structure differentially in either treatment. (Similarly, all added workers were callows, which again would not have altered the worker age-structure differentially.) Moreover, excess workers removed was found to be significantly negatively associated with queen longevity; hence, as R colonies had fewer excess workers removed, the effect of excess workers removed as a factor was counter to the prediction of H1, and could not have accounted for the independent effect of treatment in reducing overall queen longevity in R colonies.

Almond et al. [45] showed that egg removal caused an increase in worker-to-queen aggression in *B. terrestris*, which was interpreted as workers responding in a self-interested manner to a perceived loss of queen fecundity. Importantly, the experimental design of the current study differed from that of Almond et al. [45] in two key respects. First, colony size was maintained throughout at a constant level (20 workers), which may have affected workers’ response to a perceived loss of queen fecundity. Second, we removed all aggressive workers from colonies in both treatments as and when they were detected (and replaced them with callow workers) (see ‘Methods’, ‘Observed worker aggression and observed worker egg-laying’). This meant that individual workers in the current experiment would have been unable to maintain a sustained response to queen egg removal. Furthermore, Almond et al. [45] found that egg removal increased worker aggression in the pre-competition point period only, whereas the current experiment extended far into the post-competition point period (as queens had a mean longevity of 89.4 days but the first competition point occurred on day 26). Almond et al. [45] found that egg removal, though increasing worker aggression in the pre-competition point period, did not advance the competition point. In the current study, R colonies exhibited an earlier onset of worker egg-laying but not earlier worker aggression. Therefore, allowing for the differences between the two experimental designs, the results of the current study were not inconsistent with those of Almond et al. [45], and matched them in suggesting, in the given experimental contexts, a level of dissociation between the timings of workers’ egg-laying and aggression. Moreover, since R and C colonies showed no difference in worker aggression (in either overall level or time of onset), there is no evidence that differences in longevity of R and C queens were caused by differential worker treatment of them.

With respect to gene expression, R queens showed, compared to C queens, overall fewer age-related DEGs (Fig. 5) and their DEGs showed no significant overlap with genes in the TI-J-LiFe network (Fig. 6a). These results might appear inconsistent with the overall finding of support for H1, given the reduced longevity of R queens and hence the inference, on H1, that R queens experienced earlier and more rapid ageing than C queens. However, across organisms and tissues in general, it is unknown whether the number of age-related DEG scales with effects on ageing and longevity monotonically [51, 52]. In the current study system, it is instead possible that, although the number of age-related DEGs was smaller in R queens, a few key DEGs had a critical impact on longevity. For example, the TI-J-LiFe network gene *Krüppel homolog 1* was significantly downregulated with relative age in fat body in R queens but not C queens (Fig. 6a), and so represents a candidate for a single gene from this network potentially having a strong influence on queen longevity. Moreover, unlike those of C queens, DEGs in R queens showed significant overlap with the enzymatic antioxidant gene set. Overall, therefore, the transcriptomic results of the current study supported H1 and were compatible with genes in the TI-J-LiFe network and enzymatic antioxidant gene set underpinning ageing and longevity in eusocial insects as proposed [34, 36].

R and C queens in the current study also differed in both their fertility and longevity, which suggests that some of the gene expression differences between the treatments (with relative age) could have been caused by fertility differences as well as by R queens’ increased costs of reproduction affecting longevity [30, 41, 53, 54]. However, the observed treatment-specific differences in age-related gene expression, the presence of which support H1 but not H2, are unlikely to have stemmed only from the greater fertility of R queens. First, patterns of change in gene expression profiles differed between R and C queens with respect to known ageing-related genes, i.e. those in the TI-J-LiFe network [34] and the enzymatic antioxidant gene set [36]. Such differences with respect to ageing-related genes were expected under H1 but would not be expected under H2 if R and C queens differed with respect to genes differentially expressed in response to R queens’ increased egg-laying rate alone. Second, the Gene Ontology enrichment analysis showed that DEGs in R queens were not enriched specifically for processes related to reproduction, again suggesting that differences in DEGs between R and C queens were not driven only by R queens’ increased egg-laying rate. Third, although vitellogenin is an egg storage protein, a previous study of *B. terrestris* found that the gene did not change expression level with age in reproductive queens in brain or ovaries or in reproductive workers in fat body or ovaries, but it was downregulated with age in reproductive workers in brain [30]. (Age-related gene expression of vitellogenin in queen fat body was not investigated in this previous study [30].) In the current study, there was no age-related change in *vitellogenin* in brain, fat body or ovaries in R or C queens, with the exception of brain in C queens in which *vitellogenin* was downregulated with age. These results showed that R and C queens differed in age-related gene expression for this gene (and tissue), but the results were not straightforwardly attributable to R queens’ increased egg-laying rate. This is because the results in R queens for brain and ovaries and for C queens for ovaries were consistent with the previous study’s results for unmanipulated reproductive queens [30], and the results in C queens for brain and fat body were consistent with the previous study’s results for unmanipulated reproductive workers [30]. Fourth, as mentioned, R queens overall showed fewer age-related gene expression differences than C queens (Fig. 5), which is unexplained if gene expression changes were driven primarily by the increased reproduction of R queens. Lastly, if age-related gene expression changes were driven primarily by this factor, one might have expected to see such changes mainly in fat body [30, 55] and ovaries (as ovaries must be more active if egg-laying rate increases), but in fact such changes occurred mainly in brain and fat body, with very few changes occurring in ovaries (Fig. 5).

Intriguingly, there was a close similarity in gene expression profiles across treatments between queens of differing relative ages (R:TP2 queens, 60% mortality vs C:TP1 queens, 10% mortality) but similar chronological ages (85 vs 89 days old, respectively). Although there appeared not to be a common gene expression trajectory for both R and C queens, queens of almost identical chronological age showed similar gene expression profiles (Fig. 4; Additional file 1: Fig. S15) despite their different longevities and costs of reproduction. Therefore, it is possible that age-related changes in gene expression profile caused by the treatment, which were associated with the marked reduction in longevity of R queens, occurred predominantly with respect to queens’ relative age and not their chronological age. Solitary insect species do not show such invariance with chronological age [56, 57]. Hence, these results raise the possibility that *B. terrestris* queens may have undergone a partial remodelling of the genetic and endocrine networks that regulate ageing and reproduction such that the overall profile of age-related gene expression in unmanipulated queens depends more on chronological age than relative age. As described above, this conceivably represents a step in the route to the complete remodelling hypothesised to have occurred in more advanced eusocial species. However, for this possibility to be confirmed, further studies characterising age-related gene expression across a wider range of relative and chronological ages in eusocial and solitary species would need to be conducted.

## Conclusions

Experimental life-history data from *B. terrestris* support the occurrence of latent costs of reproduction to longevity in annual eusocial insects of intermediate social complexity and suggest that queens of such species exhibit condition-dependent positive fecundity-longevity associations. The accompanying gene expression profiling data also raise the possibility that some degree of remodelling of the genetic and endocrine networks underpinning ageing has occurred in intermediately eusocial species. Therefore, as others have suggested [37, 38], understanding costs of reproduction and their genetic underpinnings remains central to explaining the evolutionary trajectory of the effects of eusociality on ageing and life history.

## Methods

### Colony maintenance

We obtained 75 young *B. t. audax* colonies (mean [SD] number of workers = 9.1 [3.9]) from Biobest Group NV (Westerlo, Belgium; supplier product number: BB121040-CF2) on 28 March 2019. The queens of these colonies had been placed into hibernation on 22 October 2018 and their hibernation had ended on 18 February 2019 (Annette Van Oystaeyen, Biobest Group NV, personal communication). On receipt we transferred all colonies to wooden nest-boxes (17 × 27.5 × 16 cm) with clear Perspex lids. We kept colonies at 28 °C and 60% RH under constant red light and provided them with ad libitum sugar solution and pollen throughout the experiment. We set aside four colonies as ‘source colonies’ because either their queen had died on arrival (two colonies) or they contained more than 20 workers (two colonies). The remaining 71 ‘experimental colonies’ contained a living queen and 3–17 workers (mean 8.4 workers per colony) on receipt. We used the source colonies, supplemented by four additional source colonies obtained on 3 July 2019, to provide callow workers and brood to the 71 experimental colonies throughout the experiment. The study’s overall aim and design are summarised in Fig. 1.

To assign the experimental colonies to each treatment and to control for the effect of initial colony size (worker number) on queen fertility and longevity, we paired each colony with the colony closest to it in size, producing 35 colony pairs, and then randomly selected (using the RAND() function in Microsoft Excel) one colony from each pair for each treatment. The single unpaired colony remaining was randomly assigned to the R treatment. Hence, this procedure created 36 R (Removal) colonies and 35 C (Control) colonies. These sample sizes (allowing for the planned removal of subsets of queens for mRNA-seq) were in line with a power analysis (for detecting a difference in queen longevities of at least 10 days at power > 0.8) conducted in advance using known variances in queen longevity [16].

For the remainder of the experiment, we maintained the number of workers within each experimental colony in both R and C treatments at 20 workers, which lies within the range of sizes found in field *B. terrestris* colonies (mean [range] number of workers = 35.6 [11–75]) [58]. To achieve a constant worker number, we marked each worker initially present with white Tipp-Ex (Bic, Île-de-France, France) applied to its thorax. We then continued to mark and count workers as they eclosed every day until the colony acquired 20 workers, removing excess workers, identified as any workers without marks, after this point. Most excess workers were callows, identified from their less pigmented coats (the remainder being up to 2 days old). We used the excess callow workers together with callow workers from the source colonies to bring the number of workers in colonies with fewer than 20 workers to the required level (*B. terrestris* colonies will accept non-nestmate callow but not adult workers [59]). Once a colony had 20 workers, we continued to remove excess workers or to add callow workers to maintain worker number at 20 per colony. In addition, we removed all dead workers and, as they eclosed, all new (virgin) queens and males. In each case, to assess any effect of worker removals and deaths on queen longevity, we recorded the numbers of excess and dead workers removed from each colony and callow workers added to each colony (see ‘Results’).

### Experimental manipulation and colony fertility

To ensure that all experimental colonies could produce eggs, we defined day 1 of the experiment as the first day on which new egg cells were present in every colony across all colonies (1 April 2019). (In *B. terrestris*, eggs are laid into waxen egg cells, with several eggs being laid per cell.) To monitor new egg-cell production, we used a marker pen to map, on a clear acetate sheet fastened to the Perspex lid of the nest-box, the positions of all egg cells for each colony daily. To measure colony fertility (colony egg production including worker-laid eggs from day 1 until the last queen death on day 158), we counted the numbers of egg cells and the number of eggs within them once every 2 days (‘count days’) from each experimental colony. These included: (1) 2 count days (days 1 and 3) before the R and C treatments were started (on day 3); and (2) every count day until each queen’s death. The day 1 and 3 counts were conducted to measure queen fertility before treatments were begun. For these counts, we carefully removed and opened all new egg cells in each colony, counted the eggs in each cell, resealed the cells, and placed them back into their previous positions in each colony. From day 3, we implemented the two treatments as follows: (1) R: we removed and opened each new egg cell, counted the eggs inside, removed the eggs, resealed the empty cell, and placed it back into its previous position in the colony; (2) C: we removed and opened each new egg cell, counted the eggs inside, and, leaving the eggs in place, resealed the cell and placed it back into its previous position in the colony. This manipulation proved effective in, as intended, causing R queens to increase their egg-laying rate: from the data on during-treatment mean queen fertility (Fig. 2a), estimated egg-laying rates (mean ± SD) of R and C queens were, respectively, 52.5 ± 18.5 eggs and 25.9 ± 9.35 eggs per 48-h period. Although the manipulation therefore caused queen egg-laying rate to approximately double in R queens, it is unlikely that this exceeded the typical limits of egg production by queens. First, during treatment, over 50% of R queens had egg-laying rates within the range of egg-laying rates shown by C queens (Fig. 2a). Second, the mean egg-laying rate of R queens was within three standard deviations of that of C queens ([52.5–25.9]/9.35 = 2.8). Overall, therefore, the elevated egg-laying rates of R queens did not exceed typical limits of natural variation as shown by unmanipulated (C) queens.

We used these count data to produce two separate indices of queen fertility for each colony: (1) baseline mean queen fertility, i.e. the mean of the egg counts across each count day before experimental manipulations had begun (days 1 and 3); and (2) during-treatment mean queen fertility, i.e. the mean of the egg counts across each count day after experimental manipulations had begun up to the point when worker egg-laying was first observed (days 5–25). Some *B. terrestris* workers lay unfertilised, male eggs at a characteristic point in the colony cycle, the ‘competition point’ [60], and the first of these in any colony was observed on day 26; hence, measures of queen/colony fertility were separated into those taken before and after day 26, as the latter would have included a mixture of queen- and worker-produced eggs. As we observed no worker egg-laying in any colony before day 26, we counted all eggs laid before day 26 as queen-laid eggs (to yield baseline and during-treatment mean queen fertility). To check that our method of observing colonies for worker egg-laying at 4-day intervals (see ‘Observed worker aggression and observed worker egg-laying’, ‘Methods’) did not affect our finding that R queens showed higher egg-laying rates than C queens (see ‘Results’), we repeated our analysis comparing queen fertility in R and C colonies over days 5–21 instead of days 5–25 (there being four full days between day 21 and day 26, the day on which worker egg-laying was first observed in any colony). This showed there was a significant increase in during-treatment mean queen fertility (as measured over days 5–21) across both groups (*b* = 0.787, Seb = 0.098, *z* = 8.008, *p* < 0.001), and that this increase had a significant interaction with treatment (*b* = 0.568, Seb = 0.142, *z* = 3.997, *p* < 0.001), with R queens producing approximately twice as many eggs over days 5–21 as C queens (mean [95% CI] eggs per 48-h period: R, 47.3 [41.4, 54]; C, 25.3 [22, 29.1]). Therefore, we found that R queens had a significantly higher egg-laying rate than C queens (approximately double) regardless of whether queen fertility was measured to day 25 or day 21.

### Queen longevity

To measure queen longevity, we checked each queen daily. Once a queen had died, we recorded her date of death, removed her, and froze her and her colony at − 20 °C. Therefore, queen longevity was calculated as the number of days between day 1 and the queen’s date of death. One C queen (Q57) escaped during the count process and was therefore excluded from further analysis.

### Observed queen activity

As queen longevity was potentially affected by queens’ overall activity level, every 4 days (‘observation days’, which occurred on days without egg counts/manipulations), we recorded whether each queen was active (walking/running or engaged in egg-laying/aggression behaviours) or inactive (not engaged in these behaviours) during a brief (i.e. of a few seconds) observation period. These data were used to calculate ‘Observed queen activity’ (proportion of queens that were classified as active, in each treatment, per observation day). After the observation period, we physically carried the nest-box to a monitoring station and then recorded the queen’s response (previously active queen increases her movement speed and previously inactive queen becomes active, as defined above) or non-response (none of these events occurs). These data were used to calculate ‘response to disturbance’ (proportion of queens that responded to disturbance relative to those that did not respond to disturbance, in each treatment, per observation day).

### Observed worker aggression and observed worker egg-laying

In *B. terrestris*, removing queen eggs increases worker-to-queen aggression [45] and worker egg-laying is associated with worker-to-queen and worker-to-worker aggression [61]. We therefore sought to equalise worker aggression across R and C treatments by removing egg-laying and/or aggressive workers from all colonies [46]. For this, every 4 days, following each response to disturbance test (above), we observed the workers in each colony for 10 min. During this observation period, we recorded ‘observed worker egg-laying’ (defined as the number of worker egg-laying events observed in this period per colony per observation day) and ‘observed worker aggression’ (defined as the number of aggressive incidents directed by workers [62] towards the queen observed in this period per colony per observation day). We also recorded ‘onset of worker egg-laying’ (defined as the first day that we observed a worker egg-laying event in each colony), and ‘onset of worker aggression’ (defined as the first day that we observed a worker-to-queen aggressive incident in each colony). (The fact that, when observed, worker egg-laying events and worker aggression occurred at rates of 1–5 per colony per 10-min observation bout (Figs. 2c and 3c) suggested that our level of observation was enough to detect the onset and frequency of these behaviours sufficiently accurately. Consistent with this, these methods yielded an estimated competition point date of 37 days from first worker eclosion [26 days to first observed worker egg-laying + 11 days from first worker eclosion to day 1 of the experiment, as estimated from mean size of colonies on receipt], which matches estimates of the timing of the competition point in previous studies [16, 60, 63]). We recorded worker-to-queen but not worker-to-worker aggression as only worker-to-queen aggression was expected to have effects on queen longevity. Queen-to-worker aggression was not observed at any stage during the experiment. We also removed any egg-laying worker or aggressive (towards the queen) worker that was observed during the observation period and replaced it with a marked callow worker from a source colony or another experimental colony. Time spent removing and replacing workers was not included in the 10-min observation period.

### Digital filming

To supplement the data on queen and worker behaviour obtained from the direct observations, we also filmed each colony using Sony CDR-CX190 digital camcorders (Sony, Tokyo, Japan) under white light for 1 h on 2 days during the experiment. These 2 days (days 48 and 99) were chosen to coincide with TP1 and TP2 in the C queens (Fig. 1b; see also ‘Collection of queens for mRNA-seq’ below). The films were viewed during playback and the following metrics were quantified using the behavioural software package *BORIS* [64]: ‘filmed queen activity’ (the amount of time a queen was active during filming, per colony, per film period; activity defined as for observed queen activity); ‘filmed worker egg-laying’ (the number of worker egg-laying events recorded during filming, per colony, per film period); and ‘filmed worker aggression’ (number of worker aggressive incidents that were caught on film, per colony, per film period; aggressive incidents defined as for observed worker aggression). All film data were collected blindly with respect to colony treatment. We filmed all colonies whose queens were still alive at the time of each film period. These comprised 25 R colonies and 33 C colonies in the first film period (day 48) and 8 R colonies and 20 C colonies in the second film period (day 99). We analysed film from all R colonies (*N* = 8) filmed in both periods and a set of C colonies (*N* = 9) randomly selected from the 20 C colonies filmed in both film periods.

### Brood transfer between colonies

Because R colonies produced no larvae (as all their eggs were removed), we equalised the number of third/fourth-instar larvae and pupae across R and C colonies. We did this every 4 days (on the same days we conducted direct observations) by first removing some of the larvae and pupae from the C and source colonies. The number of these brood items removed was the maximum that could be removed without disturbing the egg cells, equating to 10–20 third/fourth-instar larvae or pupae per colony during each removal. We then divided the removed brood into two sets of equal size and placed each half into another randomly selected experimental colony. This ensured that all R and C colonies contained third/fourth-instar larvae and pupae and that these brood items had a common origin (i.e. a different C or source colony).

### Collection of queens for mRNA-seq

To prepare for sampling a subset of queens for RNA extraction, at the start of the experiment we randomly assigned each queen to two subgroups within each treatment, G1 (*N* = 20) and G2 (*N* = 15). (The additional R colony was not assigned to either subgroup or sampled for RNA.) Within each treatment, at TP1, when 10% (2/20) of the G1 queens had died, we randomly selected and removed six queens (termed TP1G queens) from the remaining G1 queens, flash froze them in liquid nitrogen and stored them at − 80 °C. Similarly, at TP2, when 60% (9/15) of G2 queens had died, we randomly sampled the remaining six queens (termed TP2G queens) in G2 in the same way. The 10% and 60% mortality thresholds were selected to represent time-points marking the occurrence of low and high mortality, respectively, during the *B. terrestris* queen lifespan. Patterns of ageing have previously been detected prior to and spanning these relative time-points in *D. melanogaster* [57, 65]. We used relative time-points [57, 66, 67], not ones based on absolute time, to account for potentially different ageing rates across the treatments (caused by differently shaped mortality curves if H1 is true) and to facilitate comparisons with other species. Hence, using this approach, we collected queens for RNA extraction at two time-points within each treatment (corresponding to the points of 10% (TP1) and 60% (TP2) queen mortality). The non-sampled G1 and G2 queens (termed life-history queens) were used to provide the queen longevity data, and all queens were used for egg count and behavioural data until they died or were sampled (Additional file 2: Table S23). Therefore, final queen sample sizes were as follows: R:TP1G (*N* = 6), R:TP2G (*N* = 6), R:life-history (*N* = 24); C:TP1G (*N* = 6), C:TP2G (*N* = 6), C:life-history (*N* = 22) (Additional file 2: Table S23).

### Tissue dissections, RNA extraction, and mRNA-seq

For each TP1G and TP2G queen, we dissected out the brain, fat body, and ovaries and flash froze all tissues in liquid nitrogen, storing each tissue from each queen individually at − 80 °C for later RNA extraction. We also measured the length of the marginal cell in each forewing (and calculated the mean marginal cell length) as an index of body size [68] in all queens (except Q57) using a Zeiss Discovery v12 Stereo microscope (Zeiss, Oberkochen, Germany) with Axiovision software (Zeiss).

For RNA extraction, we first fragmented the tissue in liquid nitrogen using a micropestle. We then added Tri-reagent (Sigma-Aldrich, Gillingham, Dorset, UK) at the level of 1 ml for each estimated 100 µg of tissue. We extracted RNA using the Direct-zol™ RNA extraction kit (Zymo Research, Irvine, CA, USA) according to the manufacturer’s protocol. We also performed an additional DNase treatment using the Turbo™ DNA-free kit (Thermo Fisher Scientific, Loughborough, UK) according to the manufacturer’s protocol. There was insufficient RNA for mRNA-seq from one TP2G brain sample in each treatment, so these two samples were excluded from further analysis. Using these procedures, we generated six biological replicates for each of the three tissues, two RNA subgroups (TP1G and TP2G), and two treatments (R and C), excepting two TP2G brain samples for which there were 5 biological replicates, leading to a total of 72 (6 × 3 × 2 × 2) minus 2 = 70 RNA samples in total (Additional file 2: Table S23). These were sent to a sequencing provider (Edinburgh Genomics) for Illumina 100 base pair, paired-end sequencing on two lanes of a NovaSeq6000 sequencer, creating two technical replicates for each biological replicate.

### Statistical analysis of fertility, life history, and behavioural data

We conducted all statistical analyses with the R (version 4.0.1) statistical programming platform in Rstudio [69], using the ‘stats’, ‘lme4’, ‘glmmTMB’, and ‘survival’ packages. We used generalised linear mixed models (glmms) to investigate the effects of treatment on queen and colony fertility, longevity, worker egg-laying, worker aggression, and queen activity. For the glmms, we created versions of each model with individual fixed effects (and random effects where appropriate) included or excluded and used the Akaike Information Criterion (AIC) to determine the final model with the best fit to the data. To determine the significance of the fixed effects of all glmms, we compared the final model with a null model (all fixed effects removed) using a likelihood ratio test (Additional file 2: Table S24). To conduct the glmms with negative binomial error distribution, we used the ‘nb.glmer’ function in lme4, and to conduct glmms with Poisson error distribution for zero-inflated count data, we used the glmmTMB function in the glmmTMB package*.* All other glmms were conducted with the lme4 package in R. We used Cox’s proportional hazards survival analysis with the ‘coxph’ and ‘coxme’ functions from the ‘survival’ and ‘coxme’ packages to determine the effect of treatment on queen longevity for the life-history queens (i.e. censoring TP1G queens, TP2G queens, and Q57 in the models) and to investigate the effects of treatment on the onset of worker egg-laying and worker aggression. For all Cox’s proportional hazards analyses, we used graphical (by plotting the Shoenfield residuals against time) and analytical tests (using the cox.zph() function in the survival package) to ensure there were no violations of the proportional hazards assumption. We also tested for outliers in the Cox’s proportional hazards analysis by calculating the deviance residuals for each observation (using the ggcoxdiagnostics() function in the ‘surv_miner’ package).

We first tested for the effect of treatment on mean queen fertility. The model consisted of a glmm with negative binomial error distribution to account for overdispersion. Treatment and a binary variable (representing the periods before and after experimental manipulations were started) were fitted as fixed effects and ID (the unique number given to each queen/colony; Additional file 2: Table S23) was fitted as a random effect. We also used a glmm with negative binomial error distribution to test the effect of treatment on colony fertility (i.e. the egg count for each colony, including worker-laid eggs, on each count day until the last queen death on day 158) as a function of time, with treatment and experimental day (fitted as a quadratic term to account for its non-linear effect on fertility) as fixed effects in the model. We created versions of the model that included random effects for ID and experimental day. The model with the lowest AIC included ID and experimental day as random effects (Additional file 2: Table S24).

To test whether colony fertility was affected by worker egg-laying, we used zero-inflated glmms with binomial error distribution to determine whether there were any differences in the levels of observed worker egg-laying between R and C colonies. The data were not found to be overdispersed. We fitted treatment and day as fixed effects and we created versions of the model that included random effects for ID and experimental day. The model was compared with and without these terms using AIC to determine the best model fit. The model with the lowest AIC included ID and day as random effects (Additional file 2: Table S24). We did not statistically analyse the number of filmed worker egg-laying events, as only nine workers were observed laying eggs in the total 32 h of analysed digital film. In addition, we used Cox’s proportional hazards survival analysis to determine the effect of treatment on the onset of worker egg-laying. We included all colonies in the model, including colonies in which worker egg-laying was not observed (in each case these were censored on the day the queen died in each colony). This model showed a decline in the regression coefficients with time, which indicated a violation of the non-proportional hazards assumption. The decline was driven by three censored R queens (Q5, Q56 and Q70) with large (< − 2) residual deviance values. The proportional hazards assumption was met when we excluded these individuals (rather than censoring them) and excluding them did not change the predictions of the model. Therefore the final Cox’s proportional hazards model of worker egg-laying that we report excluded these individuals.

We used a negative binomial glmm to determine whether the following variables (all expressed in units of numbers of workers) were significantly different between treatments to control for their potential influence on queen longevity: callow workers added, excess workers removed, dead workers removed, egg-laying workers removed, and aggressive workers removed. For each of these variables, including age (the number of days between day 1 and queen death) as a fixed effect significantly improved the model fit (lower AIC, likelihood ratio test *p* < 0.05), so it was included in the final model (Additional file 2: Table S24).

We used Cox’s proportional hazards survival analysis to determine the effect of treatment on queen longevity. Life-history queens were included in the model, and the queens sampled for RNA (TP1G and TP2G queens) and Q57 were included as censored individuals. As during-treatment mean queen fertility (Fig. 2a, b), observed worker egg-laying (Fig. 2c), excess workers removed and egg-laying workers removed were significantly different between R and C colonies, we included these effects as additional fixed effects to control for their effect on queen longevity. Although observed worker aggression was not significantly different between R and C colonies (Fig. 3c), we also included this as an additional fixed effect. To account for larger numbers of observations for older colonies, each variable was converted into a per-colony rate. The per-colony rates were calculated by summing all values for the given variable across the total number of observation periods and then dividing by the total number of observation periods for each colony. We then produced one iteration for each variant of the Cox’s proportional hazards model of queen longevity where each per-colony rate (during-treatment mean queen fertility, observed worker egg-laying, observed worker aggression, excess workers removed, and egg-laying workers removed) was included as an additional fixed effect to control for its effects on queen longevity. We also compared the model with a model in which marginal cell width was included as a fixed effect to control for the potential effect of body size on queen longevity. The model was compared with and without these terms using AIC to determine the best model fit. Independently adding excess workers removed as a fixed effect, but not the other variables (marginal cell width, during-treatment mean queen fertility, observed worker egg-laying, observed worker aggression, egg-laying workers removed), significantly improved the model fit (lower AIC value, likelihood ratio test *p* < 0.05) compared to the model with all these variables removed. Therefore, the final reported Cox’s proportional hazards model of queen longevity included treatment and excess workers removed as fixed effects and none of the other variables were included in the final model (Additional file 2: Table S24).

To further test whether queen longevity was affected by worker aggression, we used zero-inflated glmms with Poisson error distribution to determine whether there were any differences in the levels of observed worker aggression between R and C colonies. As with observed worker egg-laying, we fitted treatment and day as fixed effects and we created versions of the model that included random effects for ID, experimental day, and treatment. The model was compared with and without these terms using AIC to determine the best model fit. The model with the lowest AIC included ID and day as random effects. We also tested whether there were any differences in the levels of filmed worker aggression between the R and C colonies for which we had film data. As filmed aggression rates were low (0.5 incidences per colony per hour of digital film), we analysed filmed worker aggression as a binary index indicating whether aggression was observed/not observed during each film period in each colony. We then used a glmm with binomial error distribution to test whether treatment and film period affected the filmed worker aggression index within colonies. In this model, colony ID was fitted as a random effect; however, this model fitted the data less well (higher AIC) than the null model (the intercept-only model with all fixed effects removed; Additional file 2: Table S24). We used Cox’s proportional hazards survival analysis to determine the effect of treatment on the onset of worker aggression. We included all colonies in the model, including colonies where worker aggression was not observed (these colonies were censored on the day the queen died in each colony).

We used glmms with binomial error distributions to determine whether there were any differences in observed queen activity, which was fitted as a binary response variable. We fitted treatment and day as fixed effects and we created versions of the model that included random effects for ID and experimental day. Each model was compared with and without these terms using AIC to determine the best model fit. The model with the lowest AIC that still included treatment also included ID and day as random effects; however, this model fitted the data less well (higher AIC) than the null model (Additional file 2: Table S24). We also used glmms with binomial error distributions to determine whether there were any differences in queen response to disturbance, which was fitted as a binary response variable. We fitted treatment and day as fixed effects and we created versions of the model that included random effects for ID and experimental day. Each model was compared with and without these terms using AIC to determine the best model fit. The model with the lowest AIC included ID and day as random effects. To test whether there were any differences in filmed queen activity, we used a glmm with binomial error distribution with the number of seconds a queen spent active, relative to total number of seconds she was filmed during each film period, fitted as the response variable, and treatment and film period fitted as fixed effects. We created versions of each model that included random effects for ID and film period. The model with the lowest AIC included ID as a random effect (Additional file 2: Table S24).

Lastly, to test whether there was significant relationship between queen fertility and queen longevity within each treatment (Additional file 1: Fig. S1), we used an ANCOVA to determine the combined effects of treatment and longevity on during-treatment mean queen fertility. We used graphical and analytical tests to ensure that there were no violations of the ANCOVA assumptions of homogeneity of variance (Levene’s test, *p* = 0.115) and normality of residuals (Shapiro–Wilk test, *p* = 0.410).

### Bioinformatic analysis

#### Quality assessment of mRNA-seq reads

We used several complementary approaches to assess the quality of the mRNA-seq reads. First, we used FastQC v0.11.9 [70] to examine a range of quality measures including base quality and potential adapter contamination in each sample, with the results for each sample combined into a report for each tissue (brain, fat body, and ovaries) using the MultiQC v1.9 python library [71] with Python v3.7 [72] (Additional files 3, 4 and 5). Subsequently, we aligned reads against the *Bombus terrestris* genome (Bombus_terrestris.Bter_1.0.dna.toplevel.fa) [73] using HISAT2 v2.1.0 [74] and recorded mapping statistics (Additional file 2: Table S25). We used the HISAT2 alignment files to assess gene body coverage and junction saturation using the RSeQC v3.0.1 Python library [75] with Python v3.7. These approaches showed that some samples in each tissue had large numbers of overrepresented sequences and atypical per-sequence GC content (as assessed with FastQC), and low read alignment (6.4–69.5%) (as assessed with HISAT2). We examined several of the overrepresented sequences with BLAST (https://blast.ncbi.nlm.nih.gov/Blast.cgi) [76], using blastn against the nr/nt database, which suggested that the sequences originated from bee viruses.

To further investigate the identity of reads not mapping to the *B. terrestris* genome, we pseudoaligned reads to the Holobee database (HB_Bar_v2016.1) (https://data.nal.usda.gov/dataset/holobee-database-v20161) with Kallisto v0.46.1 [77]. This is a curated database of publicly accessioned nucleotide sequences from honeybee (*A. mellifera*) holobionts (no similar resource exists for bumblebees, which belong to the same family, the Apidae, as *Apis*). Examining the normalised read counts revealed 10 sequences, from 8 different holobionts, that had > 500 total counts in at least 2 out of the 3 tissues (Additional file 2: Tables S26—S28). Plotting the normalised counts against the percentage of reads aligned to the *B. terrestris* genome with HISAT2 for each sample in fat body revealed a negative relationship in two holobiont sequences, both from slow bee paralysis virus (SBPV) (Additional file 1: Fig. S16). These results imply that, in these libraries, large numbers of SBPV reads were present that caused low percentages of reads to align to the *B. terrestris* genome. The same trend was seen in brain and to a lesser extent in ovaries (Additional file 1: Fig. S17). Overall, SBPV viral RNA was observed in 11/23 brain samples, 12/24 fat body samples, and 12/24 ovaries samples. In each case, if a given queen showed SBPV viral RNA sequences to be present in one tissue, these sequences were also present in the other two tissues sampled, suggesting that 12 queens in total were infected with SBPV (with one such queen not yielding a brain sample that underwent mRNA-seq). Of the 12 infected queens, 4 were R queens and 8 were C queens. SBPV is found in wild *B. terrestris* [78], and, in conditions of normal feeding (as in the current study), *B. terrestris* workers experimentally infected or not infected with SBPV showed no differences in longevity [79]. The latter finding suggests that SBPV does not affect the expression of ageing-related genes in *B. terrestris*. Nonetheless, we assessed whether the presence of SBPV was likely to significantly alter gene expression in the samples by examining sample clustering, based on gene expression, using a principal component analysis (PCA). We pseudoaligned reads to the *B. terrestris* transcriptome (Bombus_terrestris.Bter_1.0.cdna.all.fa) with Kallisto v0.46.1 [77], with estimated transcript counts being summarised per gene with tximport v1.22.1 [80], followed by differential expression analysis and data visualisation using DESeq2 v1.34.0 [81]. We first assessed the variation in gene expression between technical replicates of the same biological replicate and found that technical replicates were extremely similar (data not shown). Therefore, in all subsequent analyses, we collapsed technical replicates using the collapseReplicates() function in DESeq2. The subsequent PCA plot revealed that samples in each tissue clustered by treatment and/or time-point, rather than by presence of SBPV (Additional file 1: Fig. S18), suggesting that the presence of SBPV was unlikely to significantly alter gene expression in the samples.

To further assess whether the presence of SBPV was likely to significantly alter the number and identity of DEGs, we performed differential expression analysis using DESeq2 with an FDR adjusted *p-*value threshold of 0.05 and the model ~ condition, in which condition was a categorical factor denoting the combined treatment and time-point of a sample, for two subsets of samples: these were samples that had very large numbers (85,665–22,546,361 aligned reads) of SBPV reads (termed ‘with virus’ samples), and samples that had very low numbers (3–6718 aligned reads) of SBPV reads (termed ‘no virus’ samples as very low number of virus reads were present and there was no evidence that they affected numbers of other reads aligning to the *B. terrestris* genome; Additional file 1: Fig. S17) (Additional file 2: Tables S26—S28). Differential expression analysis was conducted only when a minimum of two biological replicates for each treatment/time-point could be compared, resulting in six analyses being conducted for ‘no virus’ samples (two treatment/time-point combinations (R:TP1G vs. R:TP2G and R:TP2G vs. C:TP2G) in three tissues (brain, fat body and ovaries)) and two analyses being conducted for ‘with virus’ samples (one treatment/time-point combination (R:TP2G vs. C:TP2G) for two tissues (brain and ovaries)). The identities of the DEGs were then compared to genes differentially expressed when all the samples (‘all samples’) were analysed. Overall, numbers of DEGs were smaller in ‘no virus’ and ‘with virus’ (Additional file 1: Fig. S19), as expected when using relatively few biological replicates [82, 83]. We found a large degree of overlap between DEGs returned by ‘all samples’ and those returned by either ‘no virus’ or ‘with virus’ samples (Additional file 1: Fig. S19). Specifically, across comparisons with > 50 DEGs, ‘with virus’ samples returned a mean (range) of 7.86% (2.45–12.7%) of DEGs not returned by ‘all samples’, and ‘no virus’ samples returned a mean (range) of 3.41% (0.24–8.23%) of DEGs not returned by ‘all samples’ (Additional file 1: Fig. S19). Since these values are low, it again appeared that the presence of some queens with virus did not affect the conclusions of the mRNA-seq analysis. In addition, to address potential effects on comparisons with < 50 DEGs in this reanalysis, and to further test for any influence of virus presence, we repeated our analysis testing for overlaps in age-related DEGs between R and C queens (Fig. 5) using ‘no virus’ R samples alone. This reanalysis returned no significant overlaps in 3/6 comparisons and significant overlaps in 3/6 comparisons. (The significant overlaps were as follows: (a) in brain, 11/23 of the DEGs upregulated in R were also upregulated in C (Fisher’s exact test, *α* = 0.0167, *p* = 1.49 × 10^−9^); (b) in brain, 2/4 of the DEGs downregulated in R were also downregulated in C (Fisher’s exact test, *α* = 0.0167, *p* = 5.3 × 10^−3^); and (c) in ovaries, 6/7 of the DEGs upregulated in R were also upregulated in C (Fisher’s exact test, *α* = 0.0167, *p* = 7.38 × 10^−3^).) Between the original analysis and this reanalysis, 4/6 comparisons returned the same pattern (the 2/6 differences were that, in fat body, downregulated DEGs changed from significantly overlapping to not significantly overlapping, and, in ovaries, upregulated DEGs changed from not significantly overlapping to significantly overlapping). Overall, the finding of no significant overlaps in 3/6 comparisons and significant overlaps in 3/6 comparisons was the same as that of the analysis using all samples (see ‘Age-related gene expression’, ‘Results’).

Accordingly, we did not exclude any samples from the analysis on the basis of SBPV presence. However, we included in the gene expression analyses only samples exceeding a minimum threshold of 12 million pseudoaligned read pairs per sample (from the two technical replicates combined), so prioritising biological replication over sequencing depth as being more informative in such analyses [82, 83]. Two fat body samples (C TP2G biological replicates 4 and 6) did not meet this threshold and so we excluded them from the subsequent analysis.

Overall, a total of 68 samples met the quality threshold and were analysed, yielding final queen sample sizes for the mRNA-seq differential gene expression analysis as follows: brain: R:TP1G (*N* = 6), R:TP2G (*N* = 5); C:TP1G (*N* = 6), C:TP2G (*N* = 5); fat body: R:TP1G (*N* = 6), R:TP2G (*N* = 6); C:TP1G (*N* = 6), C:TP2G (*N* = 4); ovaries: R:TP1G (*N* = 6), R:TP2G (*N* = 6); C:TP1G (*N* = 6), C:TP2G (*N* = 6).

#### Differential gene expression between time-points within treatments

We performed differential expression analysis using DESeq2 (FDR adjusted *p-*value threshold of 0.05) and the model ~ virus + condition, in which virus was a categorical factor denoting the presence of many SBPV-aligning reads in a sample and condition was a categorical factor denoting the combined treatment and time-point of a sample. We produced boxplots of the normalised count data and principal component analysis from DESeq2 for each tissue to check normalisation and library clustering, respectively (Additional file 1: Figs. S20 – S22). This procedure generated four lists of DEGs from the differential expression analysis for each tissue: genes more highly expressed in TP2 than TP1 (upregulated genes) and genes more highly expressed in TP1 than TP2 (downregulated genes) for both R and C treatments (Additional file 2: Tables S4—S6).

#### Gene Ontology (GO) enrichment analysis

To perform GO enrichment analysis, and comparative analyses with other gene lists, we used Orthofinder v2.5.2 [84] to identify orthologues between *B. terrestris*, *D. melanogaster*, and *A. mellifera*. *D. melanogaster* single-copy orthologues for *B. terrestris* DEGs were used for GO enrichment analysis, as GO annotations for *D. melanogaster* are much more complete. GO enrichment analysis was then performed in R (v4.1.3) [69] via the clusterProfiler package (v3.16.1) [85] using biological processes GO annotations from the org.Dm.eg.db package (v3.11.4) [86]. We used an over-representation test [87] to identify GO terms that were significantly overrepresented (*p* < 0.05 after adjustment for multiple testing with *Benjamini-Hochberg*) in a set of DEGs against a background consisting of all genes that were expressed in the relevant tissue. Redundancy in the resulting enriched GO terms was reduced using the GoSemSim package (v2.14.2) [88, 89]. In ‘Results’, we report as ‘enriched’ only the significantly overrepresented non-redundant GO terms.

#### Queen age-related gene expression between treatments

We performed Fisher’s exact tests to detect significant overlaps between each pair of lists from R and C colonies for each tissue (brain, fat body, and ovaries) and each direction of differential expression with respect to age (up- and downregulated with age), resulting in six comparisons in total. The tests were performed using custom R (v4.1.3) scripts [69] adjusted for multiple testing using Bonferroni correction.

#### Comparisons with age- and ageing-related genes from other species

We compared lists of age-related genes in *B. terrestris* queens from the analysis of differential gene expression between time-points within treatments (current study) with lists of age- or ageing-related genes reported from other studies. For comparison with age-related genes, we used gene lists from two *D. melanogaster* mRNA-seq studies in which relative mortalities at sampling were comparable to those in the current study and two of the same tissues were studied, i.e. brain [50] and fat body [49]. We downloaded the total gene list and the list of genes that were differentially expressed (as defined by each study) between the appropriate time-points. Pacifico et al. [50] sampled at four time-points, so we used for the comparisons genes differentially expressed between 5-day-old and 30-day-old adults, as relative mortalities at these ages (5 days: ~ 30% mortality, 30 days: ~ 55% mortality) were very broadly similar to those in the current study. Chen et al. [49] sampled at two time-points, so we used genes differentially expressed between these points (5 days post eclosion, 50 days post eclosion). We also compared our data with results of a study showing that expression of vitellogenin increases with age in the head of *A. mellifera* queens [90]. For comparisons with ageing-related genes, we compared the DEGs from the current study to the *D. melanogaster* genes in the GenAge database [48], which catalogues experimentally validated, ageing-related genes. In addition, we compared them with gene lists from the two recent studies hypothesising that genes from the TI-J-LiFe network [34] and enzymatic antioxidant gene set [36] play a role in ageing in eusocial insects (see ‘Background’).

For all gene-list comparisons in these analyses, we identified single-copy orthologues of the relevant genes between *B. terrestris* and the comparison insect species using the OrthoFinder results (see ‘Gene Ontology (GO) enrichment analysis’, ‘Methods’). We then performed Fisher’s exact tests to detect significant overlaps between lists of DEGs from *B. terrestris* R or C queens (current study) and the lists of genes from the comparison species/studies. We compared the DEGs from Pacifico et al. [50] and Chen et al. [49] to the DEGs from *B. terrestris* R and C queens in brain and fat body (respectively), comparing between lists of genes differentially expressed in the same direction with respect to time (2 treatments × 2 tissues × 2 directions of differential expression = total of 8 comparisons). We compared the GenAge database gene list to the combined up- and downregulated genes within each treatment and tissue in the current study, as there was no expectation as to whether the GenAge database genes would be up- or downregulated with age (total 6 comparisons). We modified the approach taken by Korb et al. [34] to compare the DEGs from *B. terrestris* (current study) to the *D. melanogaster* orthologues in the TI-J-LiFe network and the enzymatic antioxidant gene set. For this, we combined up- and downregulated DEGs within each treatment and tissue in the current study and ranked them by log fold change in expression with time. We then picked out the 50 genes with the most positive log fold change and the 50 genes with the most negative log fold change within each treatment/tissue (hereafter, ‘top ± 50 genes’). Likewise, where the number of DEGs in a treatment/tissue allowed, we extracted the top ± 100 genes, top ± 200 genes, top ± 300 genes, top ± 500 genes, and all genes. We then compared the gene lists from the TI-J-LiFe network and the enzymatic antioxidant gene set to the available ‘top ± ’ genes lists for each treatment/tissue combination (total of 24 comparisons for each of the TI-J-LiFe network and the enzymatic antioxidant gene set gene lists). As before, we performed Fisher’s exact tests using custom R (v4.1.3) scripts [69] and adjusted for multiple testing using Bonferroni correction.

#### Differential gene expression comparison between time-points across treatments (i.e. comparison between relative and chronological age)

As R:TP2 and C:TP1 queens were sampled at similar chronological ages, we also compared gene expression profiles between R and C queens. We conducted differential gene expression analyses in DESeq2 using an FDR adjusted *p-*value threshold of 0.05. For the analysis of R:TP2 and C:TP1, this procedure generated two lists of DEGs within each tissue: genes more highly expressed in C:TP1 than R:TP2 (upregulated genes, i.e. increasing expression with chronological age) and genes more highly expressed in R:TP2 than C:TP1 (downregulated genes, i.e. decreasing expression with chronological age). We then repeated this procedure to compare R:TP1 with C:TP1, and R:TP2 with C:TP2. Overall, these analyses resulted in 18 gene lists (3 comparisons × 3 tissues × 2 upregulated/downregulated gene lists) (Additional file 2: Tables S18—S20). We performed Fisher’s exact tests to detect significant overlaps between each pair of gene lists that overlapped in time period. We adjusted for multiple testing using Bonferroni correction. The results showed that only 7 genes were differentially expressed between R:TP2 and C:TP1 (Fig. 4). We therefore sought to determine if these 7 DEGs were ageing-related by investigating if they occurred in the Gen Age database, TI-J-LiFe network or enzymatic antioxidant gene set genes. However, this was not possible, as these 7 DEGs proved to have no single-copy orthologues in *D. melanogaster*.

## Supplementary Information

**Additional file 1: Fig. S1.** The relationship between queen longevity and during-treatment mean queen fertility in *Bombus terrestris *queens. **Fig. S2.** Egg-cell numbers for R and C *Bombus terrestris* colonies. **Fig. S3.** Numbers of eggs per cell for experimental *Bombus terrestris* colonies. **Fig. S4.** Filmed worker egg-laying for experimental *Bombus terrestris *colonies. **Fig. S5.** Onset of worker egg-laying for experimental *Bombus terrestris *colonies. **Fig. S6.** Filmed worker aggression for experimental *Bombus terrestris* colonies. **Fig. S7.** Onset of worker aggression for experimental *Bombus terrestris *colonies. **Fig. S8.** Observed activity levels for experimental *Bombus terrestris* queens. **Fig. S9.** Filmed queen activity for experimental *Bombus terrestris* queens. **Fig. S10.** Observed response to disturbance for experimental *Bombus terrestris* queens. **Fig. S11.** Gene expression differences from mRNA-seq libraries prepared from single brain samples of *Bombus terrestris* queens. **Fig. S12.** Gene expression differences from mRNA-seq libraries prepared from single fat body samples of *Bombus terrestris* queens. **Fig. S13.** Gene expression differences from mRNA-seq libraries prepared from single ovaries samples of *Bombus terrestris* queens. **Fig. S14.** Age-related gene expression patterns compared between *Bombus terrestris *queens and the *Drosophila melanogaster* GenAge database. **Fig. S15.** MA-plots comparing gene expression profiles by chronological age in experimental *Bombus terrestris *queens. **Fig. S16.** Relationship between holobiont sequence presence in fat body and alignment to the *Bombus terrestris* genome. **Fig. S17.** Relationship between slow bee paralysis sequence presence and alignment to the *Bombus terrestris* genome. **Fig. S18.** Principal component analysis for mRNA-seq libraries from single *Bombus terrestris* queens. **Fig. S19.** Gene expression profile comparisons by relative age, treatment, and SBPV status in *Bombus terrestris *queens. **Fig. S20.** Exploratory plots from the differential gene expression analysis in brain of single *Bombus terrestris *queens. **Fig. S21.** Exploratory plots from the differential gene expression analysis in fat body of single *Bombus terrestris *queens. **Fig. S22.** Exploratory plots from the differential gene expression analysis in ovaries of single *Bombus terrestris *queens.

**Additional file 2: Table S1.** GLMM testing for the effects of treatment and time on queen fertility following R or C treatment. **Table S2.** Numbers of reads in each mRNA-seq library. **Table S3.** Numbers of read pairs that pseudoaligned to the *B. terrestris* transcriptome using Kallisto. **Table S4.** Gene expression in queen brain following R or C treatment. **Table S5.** Gene expression in queen fat body following R or C treatment. **Table S6.** Gene expression in queen ovaries following R or C treatment. **Table S7.** Statistical overlap of age-related genes between R and C treatments. **Table S8.** Overlapping genes of age-related genes between R and C treatments. **Table S9.** GO terms associated with DEGs between R and C treatments. **Table S10.** Statistical overlap in gene lists between the current study and comparable *Drosophila melanogaster* studies. **Table S11.** Overlapping genes in gene lists between current study and comparable *Drosophila melanogaster* studies; **Table S12.** Statistical overlap in age-related genes from the current study and ageing-related genes from the *Drosophila melanogaster* GenAge database. **Table S13.** Overlapping genes between current study and the *Drosophila melanogaster* GenAge database. **Table S14.** Statistical overlap in age-related genes from the current study and ageing-related genes from the TI-J-LiFe network. **Table S15.** Overlapping genes between the current study and the TI-J-LiFe network. **Table S16.** Statistical overlap between age-related genes from current study and ageing-related genes from the enzymatic antioxidant gene set. **Table S17.** Overlapping genes between the current study and the enzymatic antioxidant gene set. **Table S18.** Differentially expressed genes in queen brain between R and C treatments. **Table S19.** Differentially expressed genes in queen fat body between R and C treatments. **Table S20.** Differentially expressed genes in queen ovaries between R and C treatments. **Table S21.** Statistical overlap in age-related genes within treatment and between treatments. **Table S22.** Overlapping genes between age-related genes within treatment and between treatments. **Table S23.** Treatment group, recorded longevity, and sample sizes for each *Bombus terrestris* queen. **Table S24.** GLMM model outputs. **Table S25.** Numbers of read pairs that pseudoaligned to the *Bombus terrestris* transcriptome using HISAT2. **Table S26.** Normalised read counts from pseudoalignment of brain mRNA-seq reads against the Holobee barcode database. **Table S27.** Normalised read counts from pseudoalignment of fat body mRNA-seq reads against the Holobee barcode database. **Table S28.** Normalised read counts from pseudoalignment of ovaries mRNA-seq reads against the Holobee barcode database.

**Additional file 3.** MultiQC generated report on sample qualities for all brain libraries.

**Additional file 4.** MultiQC generated report on sample qualities for all fat body libraries.

**Additional file 5.** MultiQC generated report on sample qualities for all ovaries libraries.

## Data Availability

All data generated or analysed during this study are included in this published article, its supplementary information files and publicly available repositories. Data on life history, behaviour, morphometrics, etc., from this study are available in Collins et al. [91]. The raw mRNA-seq sequencing data have been deposited in the National Center for Biotechnology Information’s (NCBI’s) Gene Expression Omnibus (GEO) at https://www.ncbi.nlm.nih.gov/geo/ and are available under series accession number *GSE172422*. The code used for the analyses is available at https://zenodo.org/record/8014039 (10.5281/zenodo.8014039).

## Abbreviations

C: Control treatment
C:TP1: Control treatment at time-point 1
C:TP1G: Individual queens in the control treatment that were sampled at time-point 1 for mRNA-seq analysis
C:TP2: Control treatment at time-point 2
C:TP2G: Individual queens in the control treatment that were sampled at time-point 2 for mRNA-seq analysis
DEGs: Differentially expressed genes
ETA: Evolutionary theory of ageing
G1: Random subgroup of queens used for sampling TP1G within each treatment
G2: Random subgroup of queens used for sampling TP2G within each treatment
H1: Hypothesis 1
H2: Hypothesis 2
IIS: Insulin-insulin signalling
JH: Juvenile hormone
R: Egg removal treatment
R:TP1: Egg removal treatment at time-point 1
R:TP1G: Individual queens in the egg removal treatment that were sampled at time-point 1 for mRNA-seq analysis
R:TP2: Egg removal treatment at time-point 2
R:TP2G: Individual queens in the egg removal treatment that were sampled at time-point 2 for mRNA-seq analysis
SBPV: Slow bee paralysis virus
TOR: Target of rapamycin
TI-J-LiFe: TOR/IIS-JH-Lifespan and Fecundity
TP1: Time-point 1
TP2: Time-point 2
