# Supplementary material for: Costs of reproduction are present but latent in eusocial bumblebee queens
Source: BMC Biol. 2023 Jul 10;21:153. doi: 10.1186/s12915-023-01648-5 (PMC10334537; doi:10.1186/s12915-023-01648-5)
Supplement: Supplementary file 1 — Additional file 1: Fig. S1. The relationship between queen longevity and during-treatment mean queen fertility in Bombus terrestris queens. Fig. S2. Egg-cell numbers for R and C Bombus terrestris colonies. Fig. S3. Numbers of eggs per cell for experimental Bombus terrestris colonies. Fig. S4. Filmed worker egg-laying for experimental Bombus terrestris colonies. Fig. S5. Onset of worker egg-laying for experimental Bombus terrestris colonies. Fig. S6. Filmed worker aggression for experimental Bombus terrestris colonies. Fig. S7. Onset of worker aggression for experimental Bombus terrestris colonies. Fig. S8. Observed activity levels for experimental Bombus terrestris queens. Fig. S9. Filmed queen activity for experimental Bombus terrestris queens. Fig. S10. Observed response to disturbance for experimental Bombus terrestris queens. Fig. S11. Gene expression differences from mRNA-seq libraries prepared from single brain samples of Bombus terrestris queens. Fig. S12. Gene expression differences from mRNA-seq libraries prepared from single fat body samples of Bombus terrestris queens. Fig. S13. Gene expression differences from mRNA-seq libraries prepared from single ovaries samples of Bombus terrestris queens. Fig. S14. Age-related gene expression patterns compared between Bombus terrestris queens and the Drosophila melanogaster GenAge database. Fig. S15. MA-plots comparing gene expression profiles by chronological age in experimental Bombus terrestris queens. Fig. S16. Relationship between holobiont sequence presence in fat body and alignment to the Bombus terrestris genome. Fig. S17. Relationship between slow bee paralysis sequence presence and alignment to the Bombus terrestris genome. Fig. S18. Principal component analysis for mRNA-seq libraries from single Bombus terrestris queens. Fig. S19. Gene expression profile comparisons by relative age, treatment, and SBPV status in Bombus terrestris queens. Fig. S20. Exploratory plots from the differential gene expression an [file 12915_2023_1648_MOESM1_ESM.docx]

Costs of reproduction are present but latent in eusocial bumblebee queens

**David H. Collins^1*^, David C. Prince^1^, Jenny L. Donelan^1^, Tracey Chapman^1^, and Andrew F. G. Bourke^1^**

^1^School of Biological Sciences, University of East Anglia, Norwich Research Park, Norwich NR4 7TJ, UK

*Correspondence: [David.Collins@uea.ac.uk](mailto:David.Collins@uea.ac.uk)

# Supplementary figures

### Fig. S1


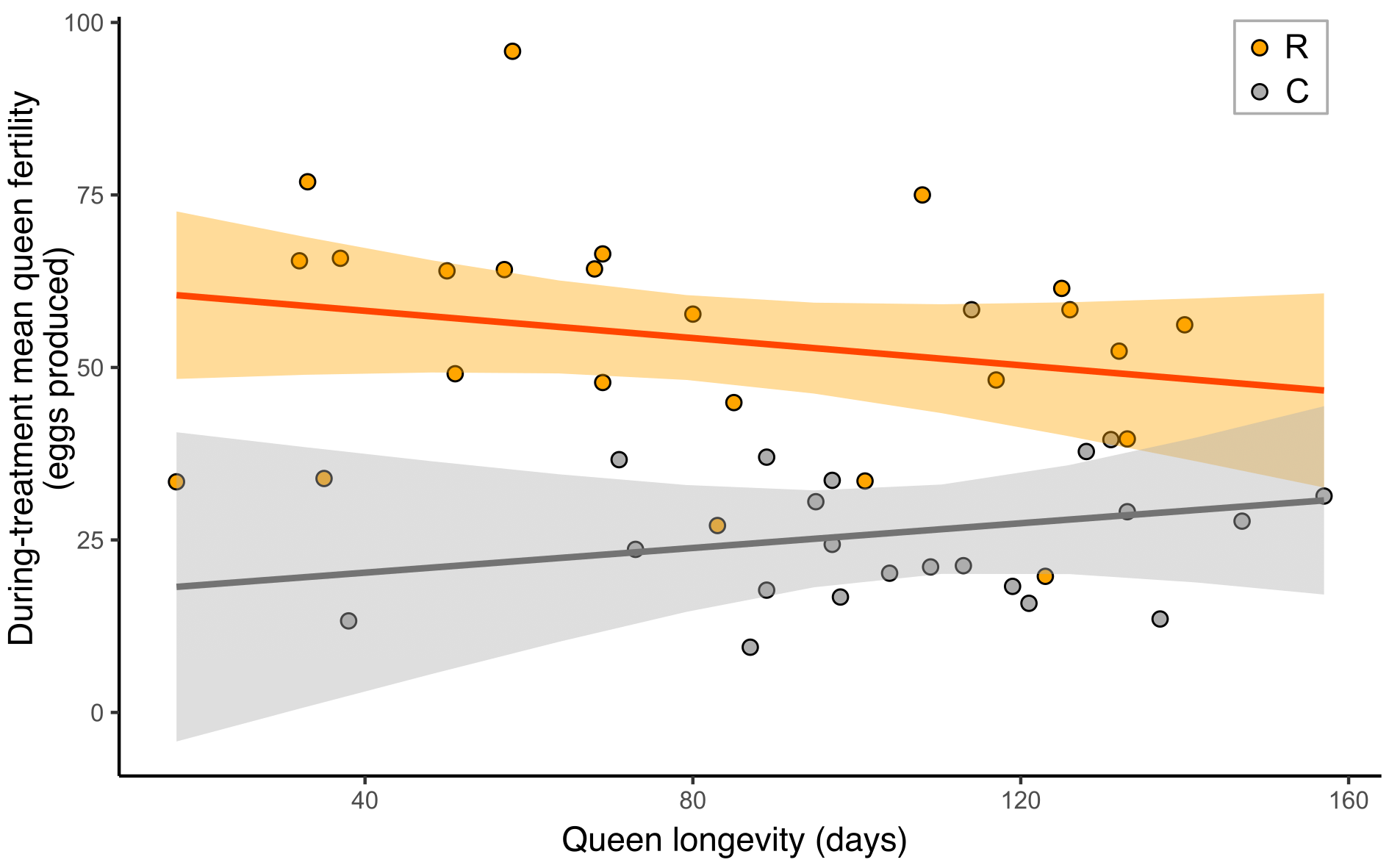


Fig. S1: The relationship between queen longevity and during-treatment mean queen fertility in *Bombus terrestris* queens. Queen longevity, number of days between day 1 of the experiment and the queen’s date of death for R (eggs removed; N = 24) and C (eggs removed and replaced; N = 22) queens; during-treatment mean queen fertility, mean number of eggs laid by queens per 48-hour period between days 5-25 inclusive. Regression line (and shading), the model fit (and confidence intervals) from the ANCOVA analysis of these data (see *Statistical analysis of fertility, life-history, and behavioural data,* main text). There was no significant relationship between fertility and longevity in either treatment (ANCOVA: R: F = 0.669, df = 1, 21, p = 0.423; C: F = 0.868, df = 1, 19, p = 0.363) and no evidence of an interaction of treatment and fertility in their effects on longevity across all queens (i.e. the slopes for R and C queens were not significantly different; ANCOVA: F = 0.367, df = 1, 40, p = 0.548). Although the relationships were not statistically significant, there was a tendency for R and C queens to exhibit a negative and positive fertility-longevity relationship, respectively.

### Fig. S2

Fig. S2: Egg-cell numbers for R (eggs removed) and C (eggs removed and replaced) *Bombus terrestris* colonies. Total number of egg cells over time for R (N = 36 on day 1 declining to N = 1 on day 134) and C (N = 35 on day 1 declining to N = 3 on day 134) colonies until day 134. Red dashed line: day 5, manipulations started; black dashed line: day 26, when worker egg-laying first observed in any colony. Daily sample sizes are in Additional file 2: Table S23. Outliers are not shown. Black horizontal bars: medians; boxes: interquartile ranges; whiskers: 1.5 × interquartile range. Over the course of the experiment, R colonies had significantly higher egg-cell numbers than C colonies (b = 1.107, SEb = 0.110, z = 19.690, p < 0.001).

### Fig. S3

Fig. S3: Numbers of eggs per cell for experimental *Bombus terrestris* colonies. Mean number of eggs per egg cell over time for R (eggs removed; N = 36 on day 1 declining to N = 1 on day 134) and C (eggs removed and replaced; N = 35 on day 1 declining to N = 3 on day 134) colonies until day 134. Red dashed line: day 5, manipulations started; black dashed line: day 26, when worker egg-laying first observed in any colony. Daily sample sizes are in Additional file 2: Table S23. Outliers are not shown. Black horizontal bars: medians; boxes: interquartile ranges; whiskers: 1.5 × interquartile range.

### Fig. S4


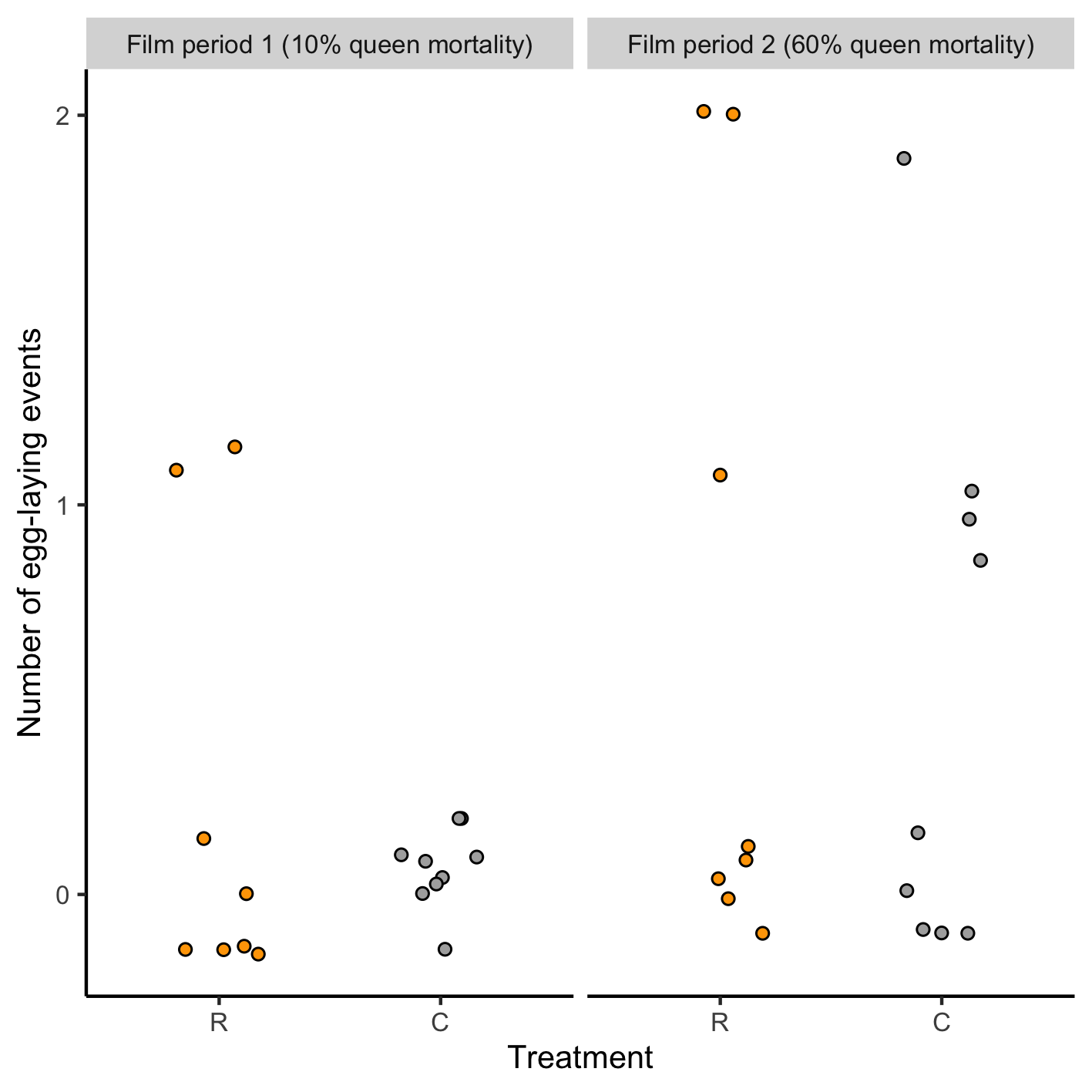


Fig. S4: Filmed worker egg-laying for experimental *Bombus terrestris* colonies. Number of egg-laying events in R (eggs removed, N = 8), and C (eggs removed and replaced, N = 9) colonies during 2 × 1-hour film periods, i.e. film period 1 on day 48 and film period 2 on day 99, to coincide with time-point 1 (TP1, 10% queen mortality) and time-point 2 (TP2, 60% queen mortality) of the C colonies, respectively. Points are offset around each integral value on both axes. Due to low sample sizes returned by the filmed worker egg-laying, these data could not be analysed statistically. Data from observed worker egg-laying showed that R colonies had significantly higher levels of worker egg-laying (Fig. 2c).

### Fig. S5

Fig. S5: Onset of worker egg-laying for experimental *Bombus terrestris* colonies. Proportion, proportion of either R (eggs removed, N = 36) or C (eggs removed and replaced, N = 35) colonies in which workers had not yet been observed egg-laying by a given time (days on the x-axis). Small vertical lines, censored queens (queens that died before egg-laying was observed). Three colonies were not included in final model (Q5, Q56, and Q70; see Methods, main text, for details). R colonies had significantly earlier onset of worker egg-laying (see Results, main text, for details).

### Fig. S6


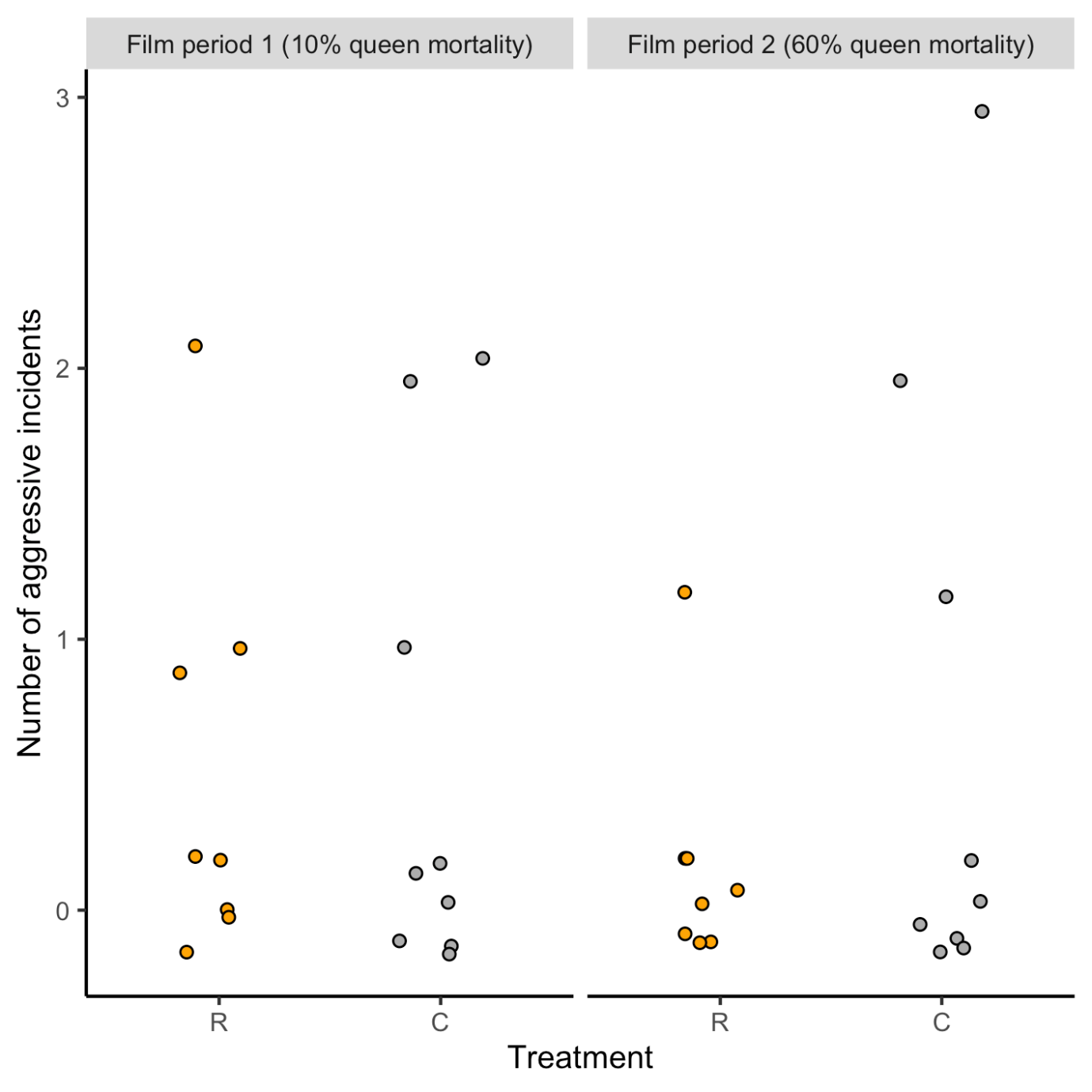


Fig. S6: Filmed worker aggression for experimental *Bombus terrestris* colonies. Number of worker-to-queen aggressive incidents in R (eggs removed, N = 8), and C (eggs removed and replaced, N = 9) during 2 × 1-hour film periods. Film periods were as defined in the Fig. S4 legend. Points are offset around each integral value on both axes. There was no significant difference in filmed worker aggression between R and C colonies (see Results, main text, for details).

### Fig. S7

Fig. S7: Onset of worker aggression for experimental *Bombus terrestris* colonies. Treatments were R (eggs removed, N = 36) and C (eggs removed and replaced, N = 35). Proportion, proportion of colonies where workers had not yet been observed egg-laying by a given time (days on the x-axis). Small vertical lines, censored queens (queens that died before aggression was observed). There was no significant difference in the onset of worker aggression between R and C colonies (see Results, main text, for details).

### Fig. S8


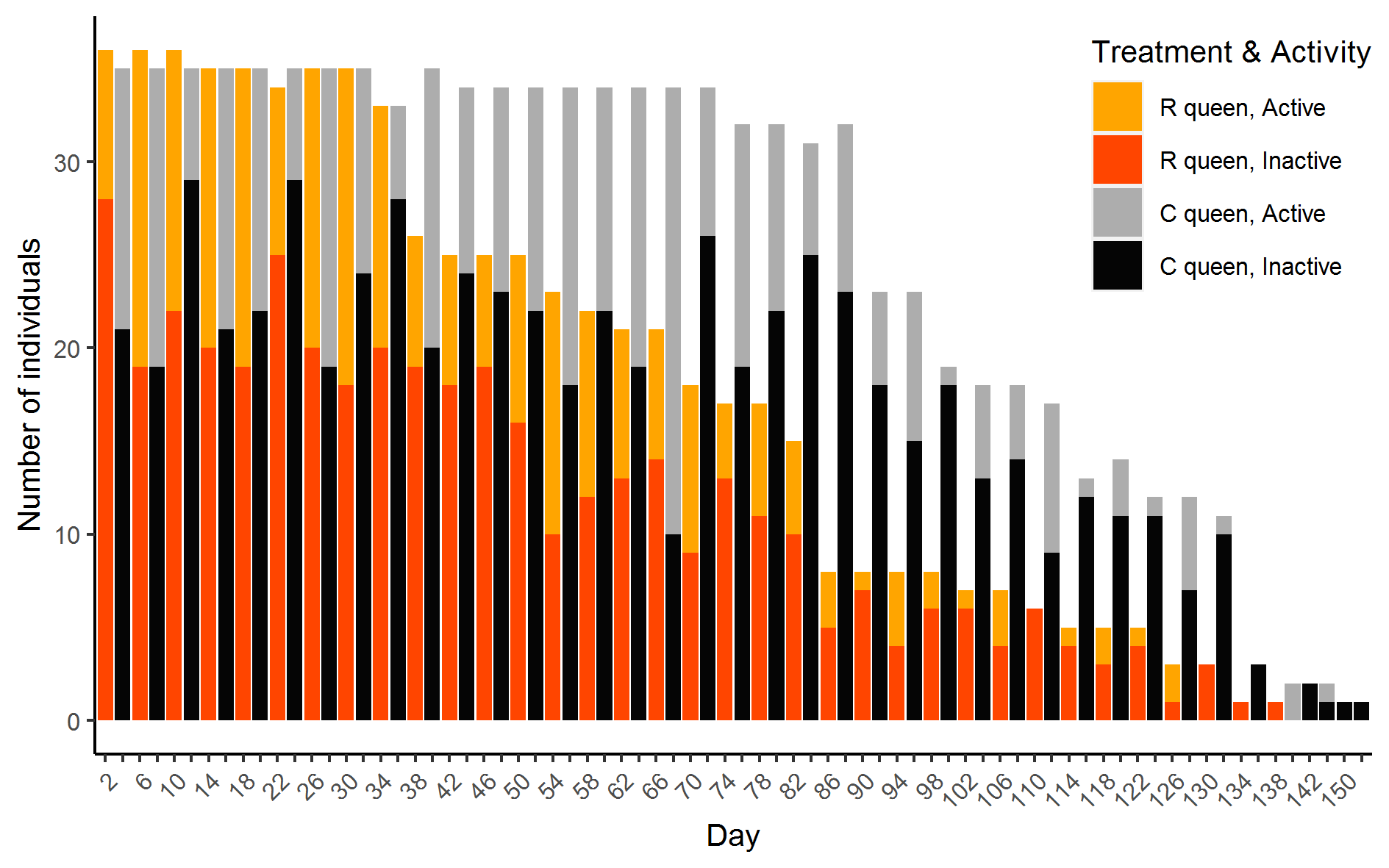


Fig. S8 Observed activity levels for experimental *Bombus terrestris* queens. Bar height: number of R (eggs removed, pale/dark orange bars, N = 36 on day 1 declining to N = 1 on day 134) or C (eggs removed and replaced, pale/dark gray bars, N = 35 on day 1 declining to N = 1 on day 148) queens alive on each day. Pale relative to dark colour (within each bar): proportion of queens that were active (moving when recorded) to inactive (not moving when recorded) on each day within each treatment. There was no significant difference in observed activity levels between R and C queens (see Results, main text, for details). In addition, the proportion of time spent active did not change throughout the experiment in either treatment (binomial glmm: b = -0.056, SEb = 0.110, z = -0.510, p = 0.610).

### Fig. S9


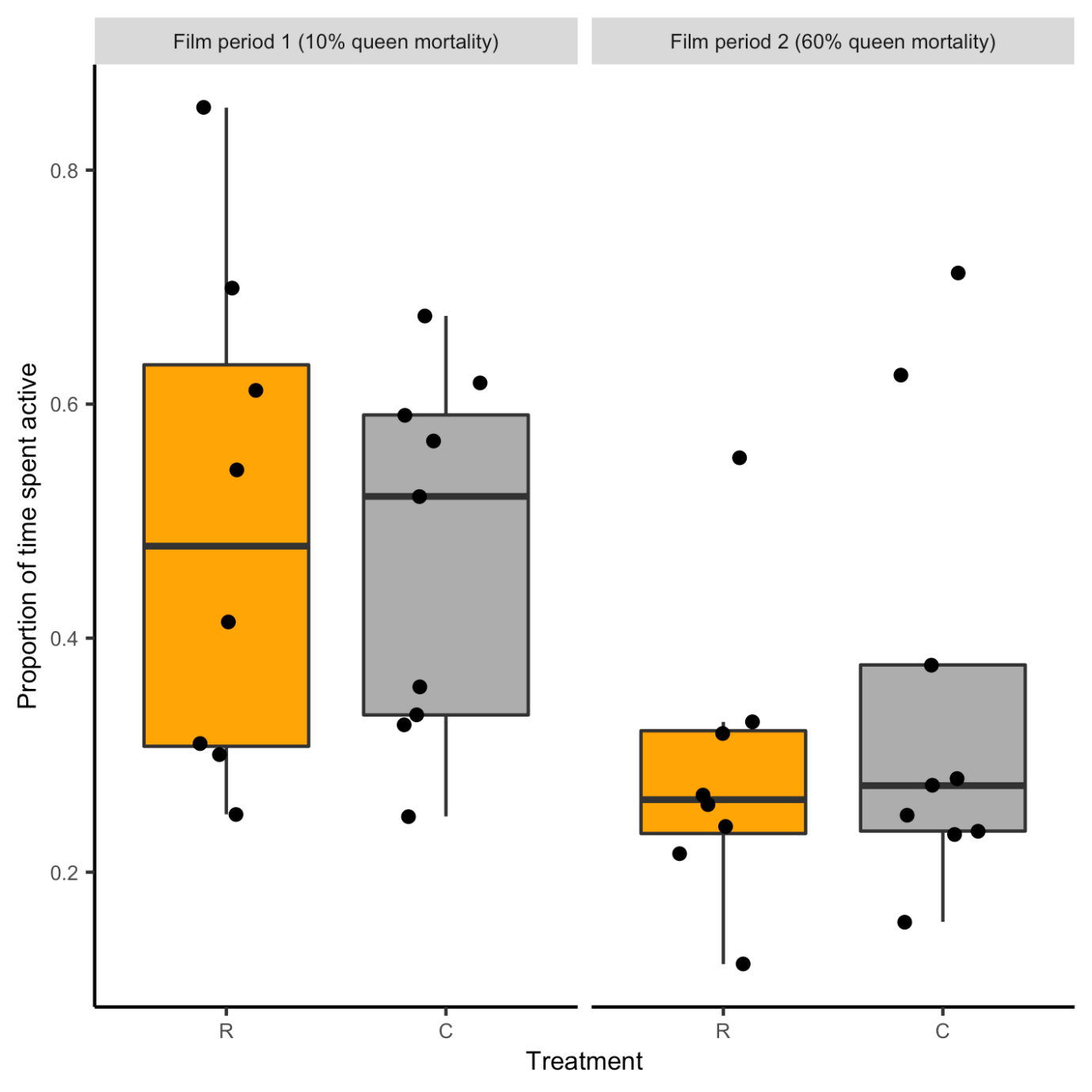


Fig. S9. Filmed queen activity for *Bombus terrestris* queens. Proportion of time spent active for R (eggs removed; N = 8) and C (eggs removed and replaced; N = 9) queens during 2 × 1-hour film periods. Film periods were as defined in the Fig. S4 legend. Black circles: individual data values; Black horizontal bars: medians; boxes: interquartile ranges; whiskers: 1.5 × interquartile range. The amount of filmed activity was significantly lower in the second than in the first period in both treatments (binomial glmm: b = -0.922, SEb = 0.019, z = -48.830, p < 0.001). However, there was no significant difference in filmed queen activity levels between R and C colonies independently of period (see Results, main text, for details).

### Fig. S10


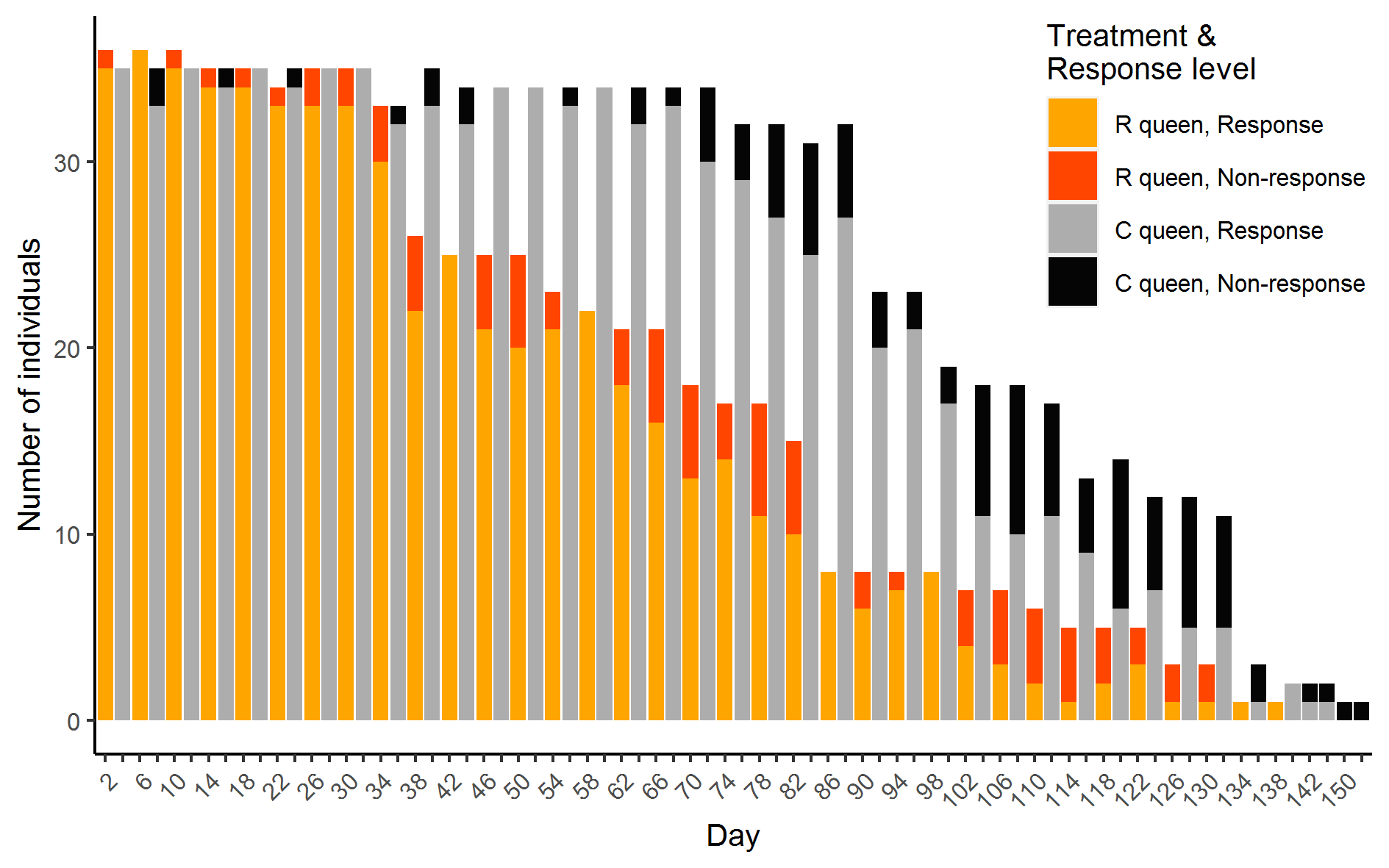


Fig. S10. Observed response to disturbance for experimental *Bombus terrestris* queens. Bar height: number of R (eggs removed, pale/dark orange bars, N = 36 on day 1 declining to N = 1 on day 134) or C (eggs removed and replaced, pale/dark gray bars, N = 35 on day 1 declining to N = 1 on day 148) queens alive on each day. Pale relative to dark colour (within each bar): proportion of queens that showed a response to disturbance (became active/increased speed after the colony was moved) to queens that showed a non-response to disturbance (did not become active/increase speed after the colony was moved) on each day within each treatment. The proportion of responses to disturbance declined significantly with time for both treatments (binomial glmm: b = -1.7963, SEb = 0.307, z = -5.856, p < 0.001). However, there was no significant difference in queens’ response to disturbance between R and C colonies (see Results, main text, for details).

### Fig. S11

**
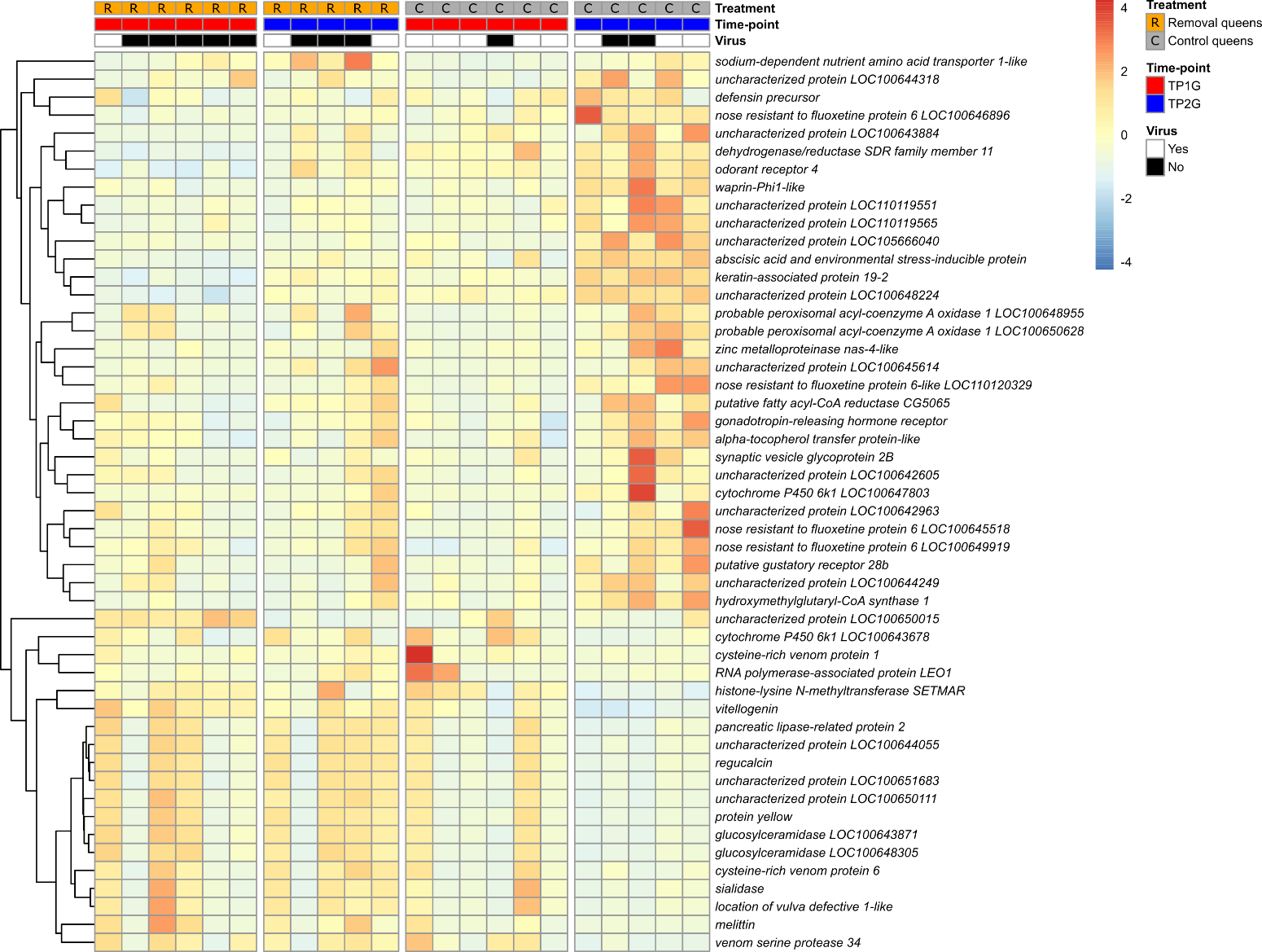
**

Fig. S11. Gene expression differences from mRNA-seq libraries prepared from single brain samples of *Bombus terrestris* queens. Differences between R (eggs removed) and C (eggs removed and replaced) queens at two time-points (TP1G and TP2G) expressed in a heatmap showing relative changes in gene expression (log_2_ fold change) within each gene for the 50 most highly differentially expressed genes (DEGs) (out of 836 DEGs in total), with each row representing an individual gene and each column representing a biological replicate of the mRNA-seq data. Vertical breaks separate the two treatments and the two time-points. The presence of virus reads in the mRNA-seq library is annotated by white (Yes – 'with virus' samples) and black (No – 'no virus' samples) bars. The dendrogram at left groups genes that cluster according to their gene expression patterns. Sample sizes: R:TP1G (N=6), R:TP2G (N=5); C:TP1G (N=6), C:TP2G (N=5).

### Fig. S12

**
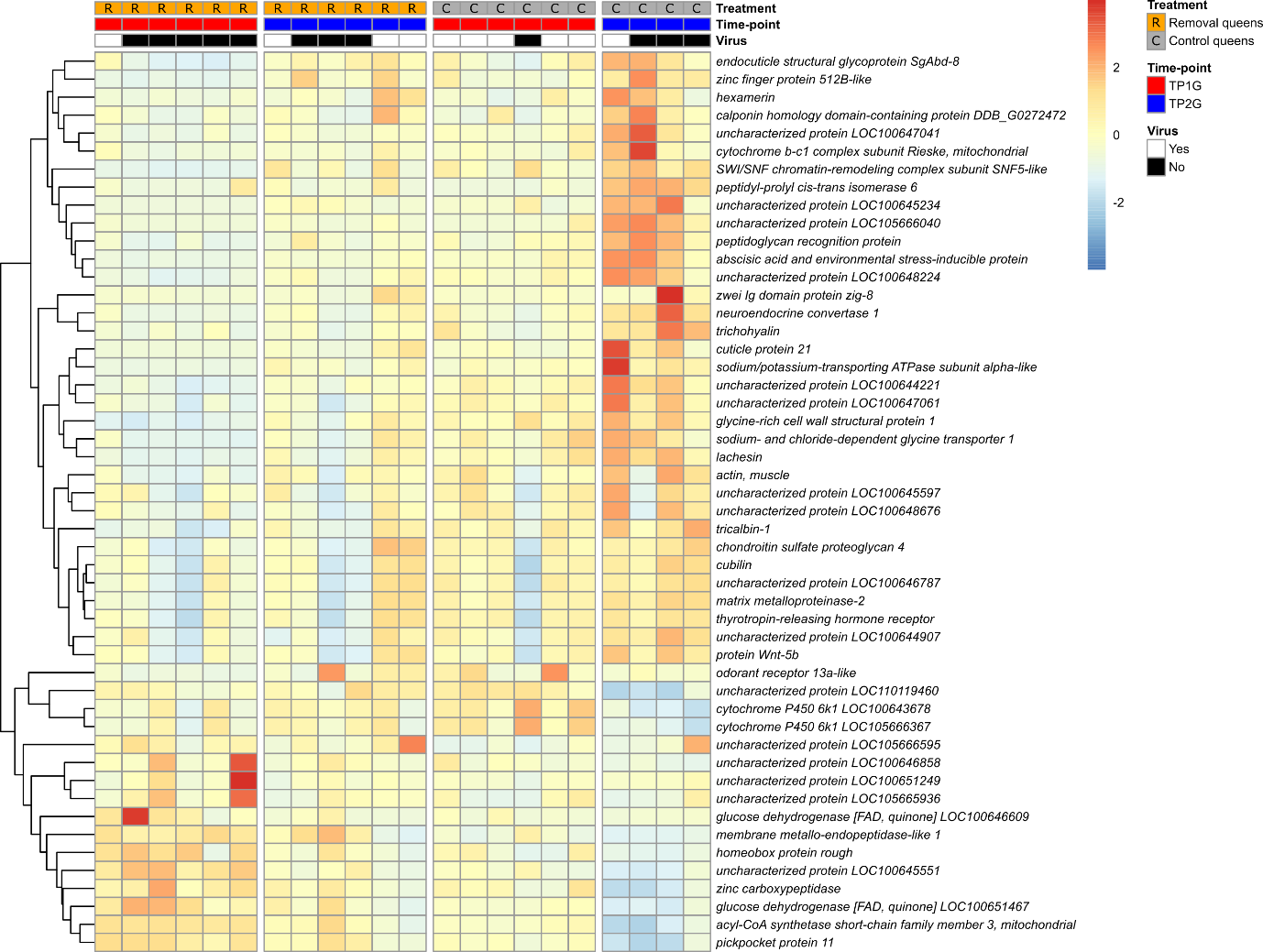
**

Fig. S12. Gene expression differences from mRNA-seq libraries prepared from single fat body samples of *Bombus terrestris* queens. Differences between R (eggs removed) and C (eggs removed and replaced) queens at two time-points (TP1G and TP2G) expressed in a heatmap showing relative changes in gene expression (log_2_ fold change) within each gene for the 50 most highly differentially expressed genes (DEGs) (out of 2,572 DEGs in total), with each row representing an individual gene and each column representing a biological replicate of the mRNA-seq data. Vertical breaks separate the two treatments and the two time-points. The presence of virus reads in the mRNA-seq library is annotated by white (Yes – 'with virus' samples) and black (No – 'no virus' samples) bars. The dendrogram at left groups genes that cluster according to their gene expression patterns. Sample sizes: R:TP1G (N=6), R:TP2G (N=6); C:TP1G (N=6), C:TP2G (N=4).

### Fig. S13


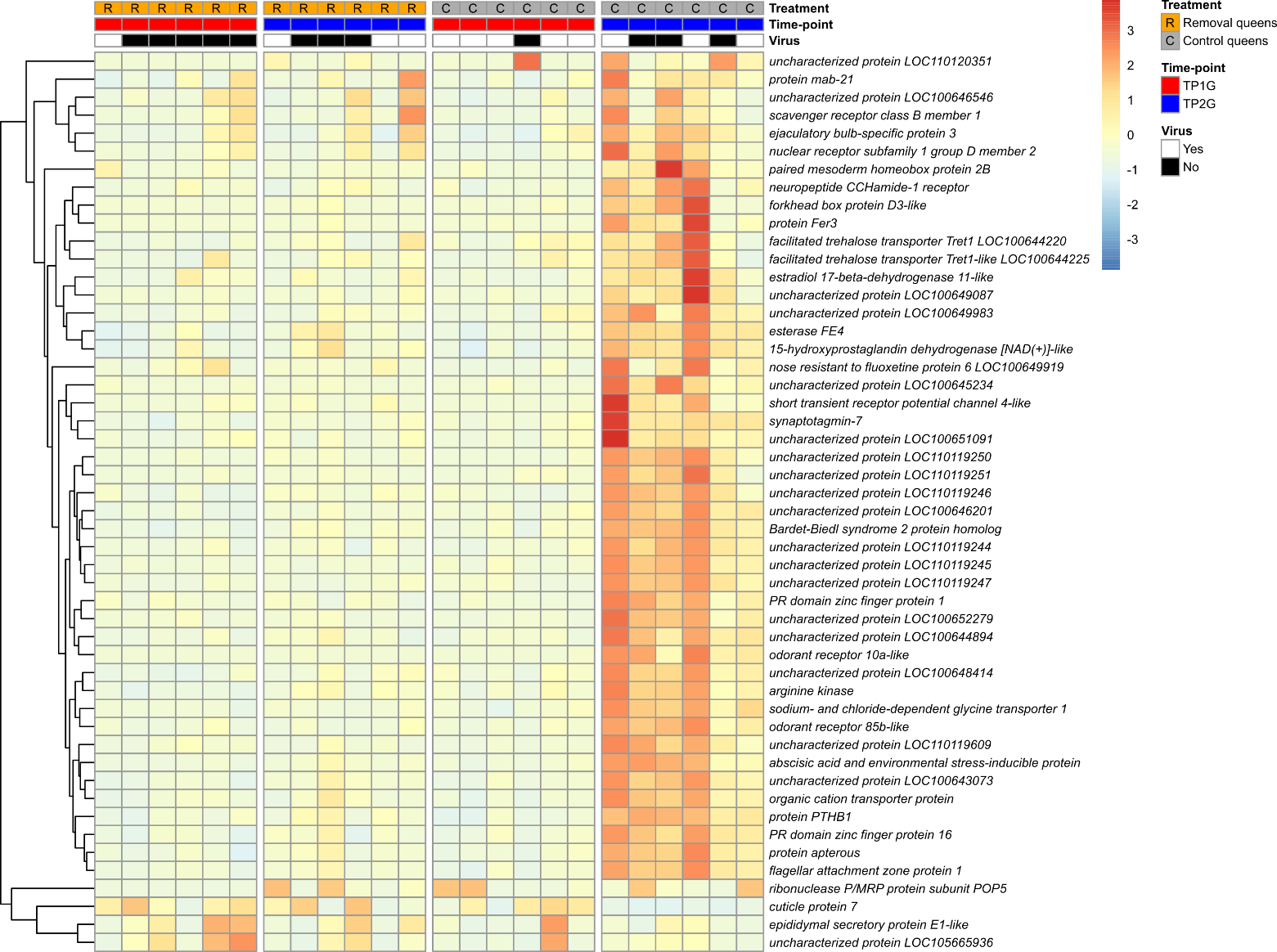


Fig. S13. Gene expression differences from mRNA-seq libraries prepared from single ovaries samples of *Bombus terrestris* queens. Differences between R (eggs removed) and C (eggs removed and replaced) queens at two time-points (TP1G and TP2G) expressed in a heatmap showing relative changes in gene expression (log_2_ fold change) within each gene for the 50 most highly differentially expressed genes (DEGs) (out of 6,437 DEGs in total), with each row representing an individual gene and each column representing a biological replicate of the mRNA-seq data. Vertical breaks separate the two treatments and the two time-points. The presence of virus reads in the mRNA-seq library is annotated by white (Yes – 'with virus' samples) and black (No – 'no virus' samples) bars. The dendrogram at left groups genes that cluster according to their gene expression patterns. R:TP1G (N=6), R:TP2G (N=6); C:TP1G (N=6), C:TP2G (N=6).

### Fig. S14


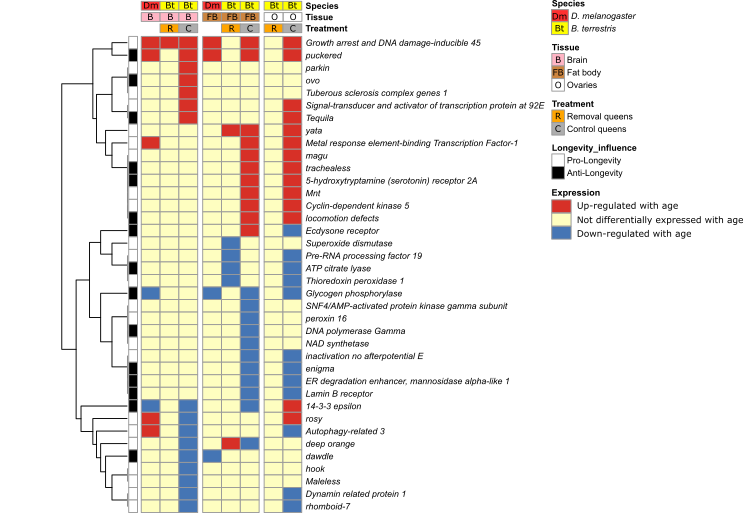


Fig. S14. Age-related gene expression patterns compared between *Bombus terrestris* queens and the *Drosophila melanogaster* GenAge database. The *D. melanogaster* GenAge database contains pro- and anti-longevity genes but does not specifiy patterns of age-related gene expression; therefore data on age-related gene expression from tissue-specific *D. melanogaster* studies (brain = Pacifico et al. (Pacifico et al. 2018), fat body = Chen et al. (Chen, Zheng, and Zheng 2014) are included in the figure for comparison. Data on expression in *B. terrestris* ovaries from the current study are shown for illustration, although no comparable data were available on *D. melanogaster* age-related gene expression in ovaries. Each row represents an individual gene showing age-related differential expression in brain or fat body of *B. terrestris* (current study) and having a single-copy orthologue present in the *D. melanogaster* GenAge database as determined by Orthofinder (N=38 unique genes from the 46 overlapping genes in Additional file 2: Tables S12, S13). Each column denotes whether a gene shows age-related differential expression in a given data set (brain: *D. melanogaster* = Pacifico et al. (Pacifico et al. 2018), *B. terrestris* = current study; fat body: *D. melanogaster* = Chen et al. (Chen, Zheng, and Zheng 2014), *B. terrestris* = current study; ovaries: *B. terrestris* = current study). Vertical breaks separate the three tissues (brain, fat body, and ovaries) and the dendogram at left groups genes that cluster together according to their gene expression patterns. Treatment: R, removal queens, eggs removed; C, control queens, eggs removed and replaced. Sample sizes in current study: brain: R:TP1G (N=6), R:TP2G (N=5); C:TP1G (N=6), C:TP2G (N=5); fat body: R:TP1G (N=6), R:TP2G (N=6); C:TP1G (N=6), C:TP2G (N=4); ovaries: R:TP1G (N=6), R:TP2G (N=6); C:TP1G (N=6), C:TP2G (N=6).

### Fig. S15


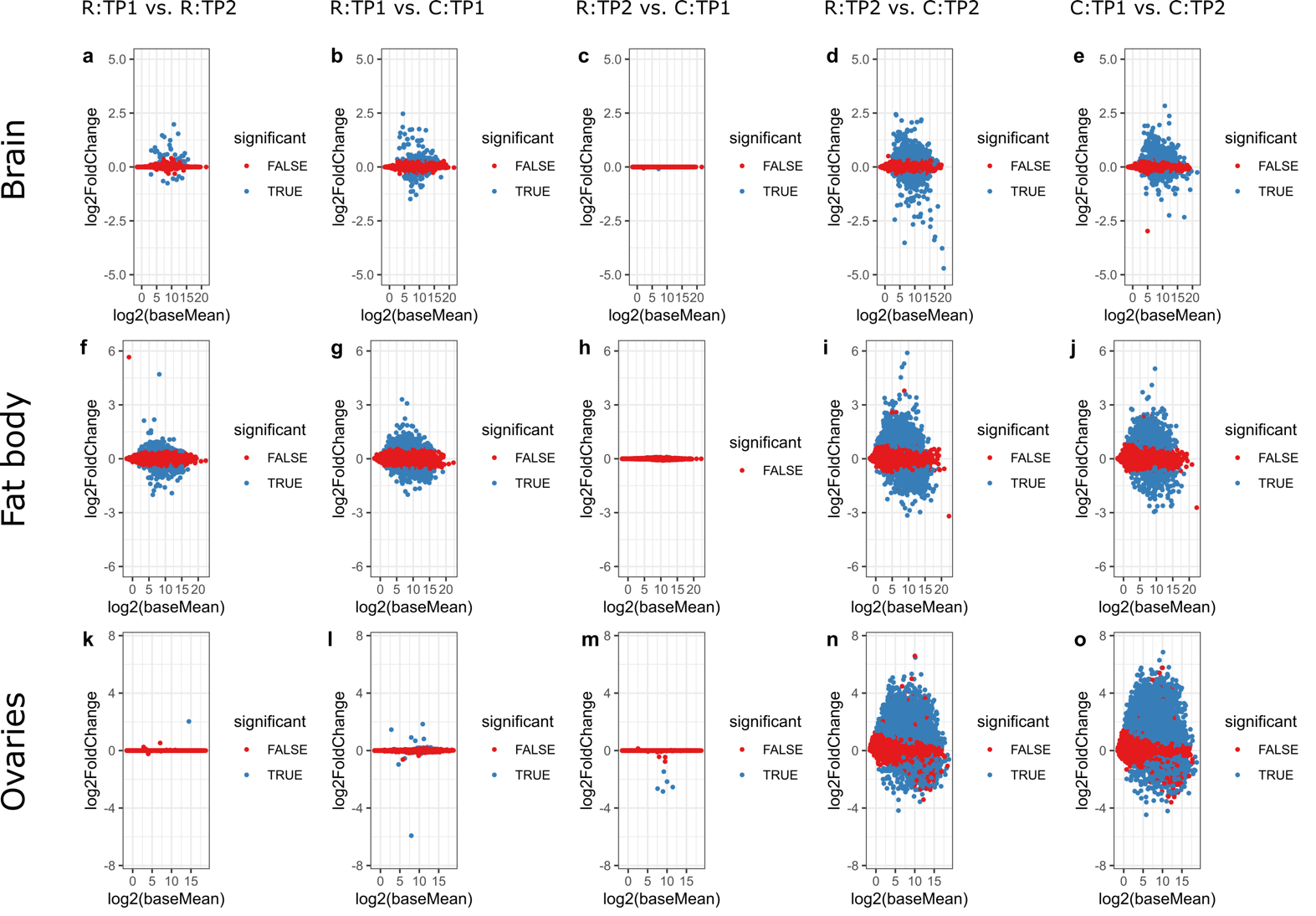


Fig. S15. MA-plots comparing gene expression profiles by chronological age in experimental *Bombus terrestris* queens. Comparisons are between pairs of treatments (R, eggs removed; C, eggs removed and replaced) and time-points (TP1 and TP2) from mRNA-seq libraries from three tissues of *Bombus terrestris* queens. X axis: log_2_ value of the mean expression of each gene in the experiment; Y axis: log_2_ value of the fold-change of expression between two samples (i.e. log_2_ of the ratio between expression of one sample and the other sample). **a-e,** brain; **f-j,** fat body; and **k-o,** ovaries. **a, f,** and **k**: comparisons between R:TP1 and R:TP2; **b, g** and **l**: comparisons between R:TP1 and C:TP1; **c, h** and **m**: comparisons between R:TP2 and C:TP1; **d, i** and **n**: comparisons between R:TP2 and C:TP2; **e, j** and **o**: comparisons between C:TP1 and C:TP2. Blue points ('true'): differentially expressed genes (DEGs); red points ('false'): genes that are not differentially expressed, based on DESeq2 analysis. Sample sizes in current study: brain: R:TP1G (N=6), R:TP2G (N=5); C:TP1G (N=6), C:TP2G (N=5); fat body: R:TP1G (N=6), R:TP2G (N=6); C:TP1G (N=6), C:TP2G (N=4); ovaries: R:TP1G (N=6), R:TP2G (N=6); C:TP1G (N=6), C:TP2G (N=6).

### Fig. S16


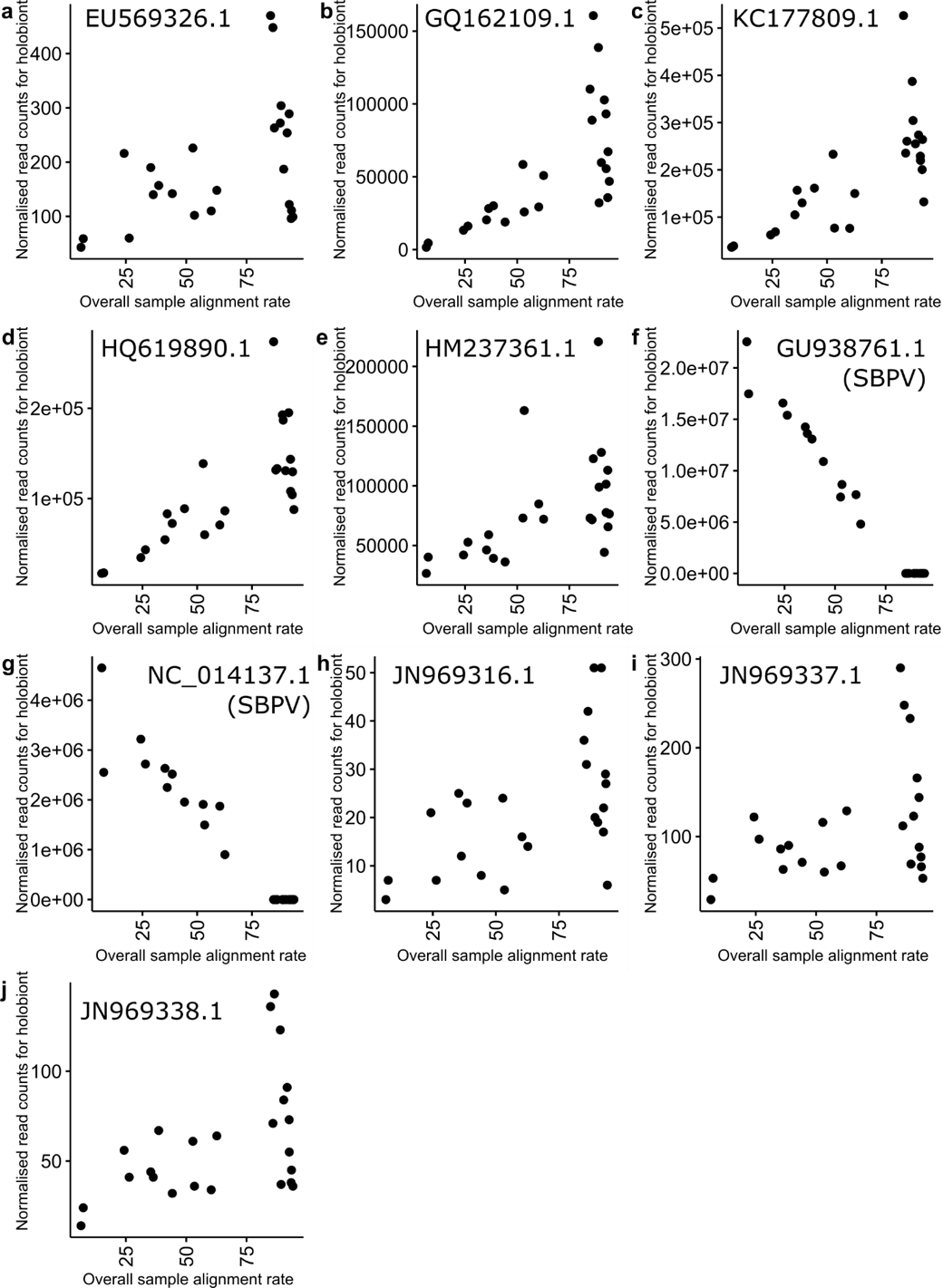


Fig. S16. Relationship between holobiont sequence presence in fat body and alignment to the *Bombus terrestris* genome. The 10 sequences shown are those with >500 normalised counts in total from Kallisto across all the libraries from a tissue, in at least 2 out of the 3 tissues (brain, fat body, and ovaries). **a-j,** Scatterplots show fat body mRNA-seq libraries from the current study (N = 24) with normalised read counts from Kallisto for an *Apis mellifera* holobiont sequence (GenBank accession given at the top of the panel), plotted against the overall percentage alignment of the mRNA-seq library to the *B. terrestris* genome using HISAT2. Holobiont sequences in panels **f** and **g** are GenBank accessions for slow bee paralysis virus (SBPV) and both show a negative relationship with overall alignment rate.

### Fig. S17


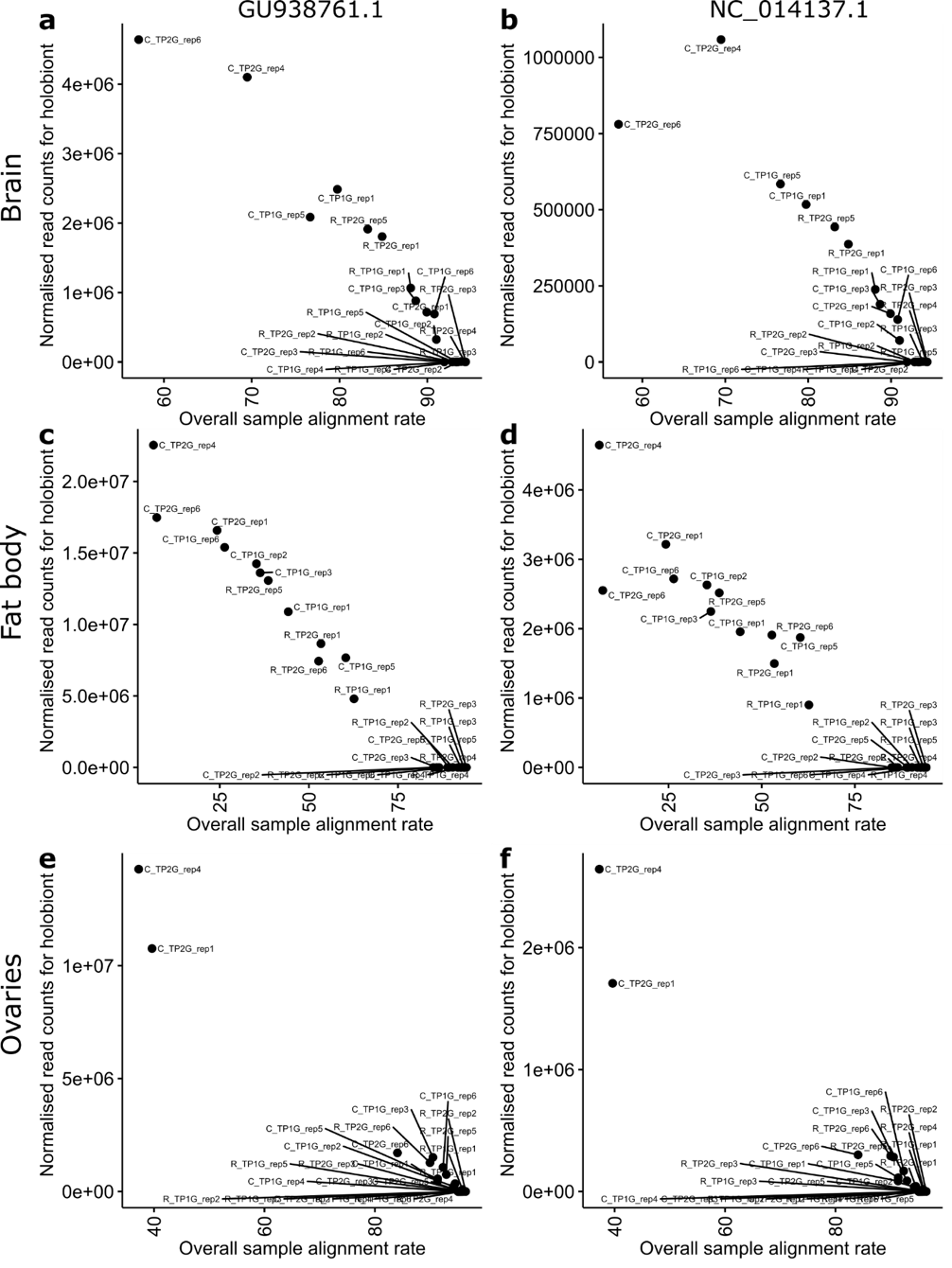


Fig. S17. Relationship between slow bee paralysis sequence (SBPV) presence and alignment to the *Bombus terrestris* genome. Scatterplots show mRNA-seq libraries from the current study with normalised read counts from Kallisto for slow bee paralysis sequences (**a, c, e,** GU938761.1 or **b, d, f,** NC_014137.1) plotted against the overall percentage alignment of the mRNA-seq library to the *Bombus terrestris* genome using HISAT2 for libraries from **a, b,** brain, N = 22, **c, d,** fat body, N = 24 or **e, f,** ovaries, N = 24. Libraries are labelled with the library name (with, if required, a black line connecting the library name and the relevant point). Library names are in the format, treatment_time-point_biological replicate. R, removal queens (eggs removed); C, control queens (eggs removed and replaced); TP1G, time-point 1; TP2G, time-point 2; rep1, biological replicate 1.

### Fig. S18


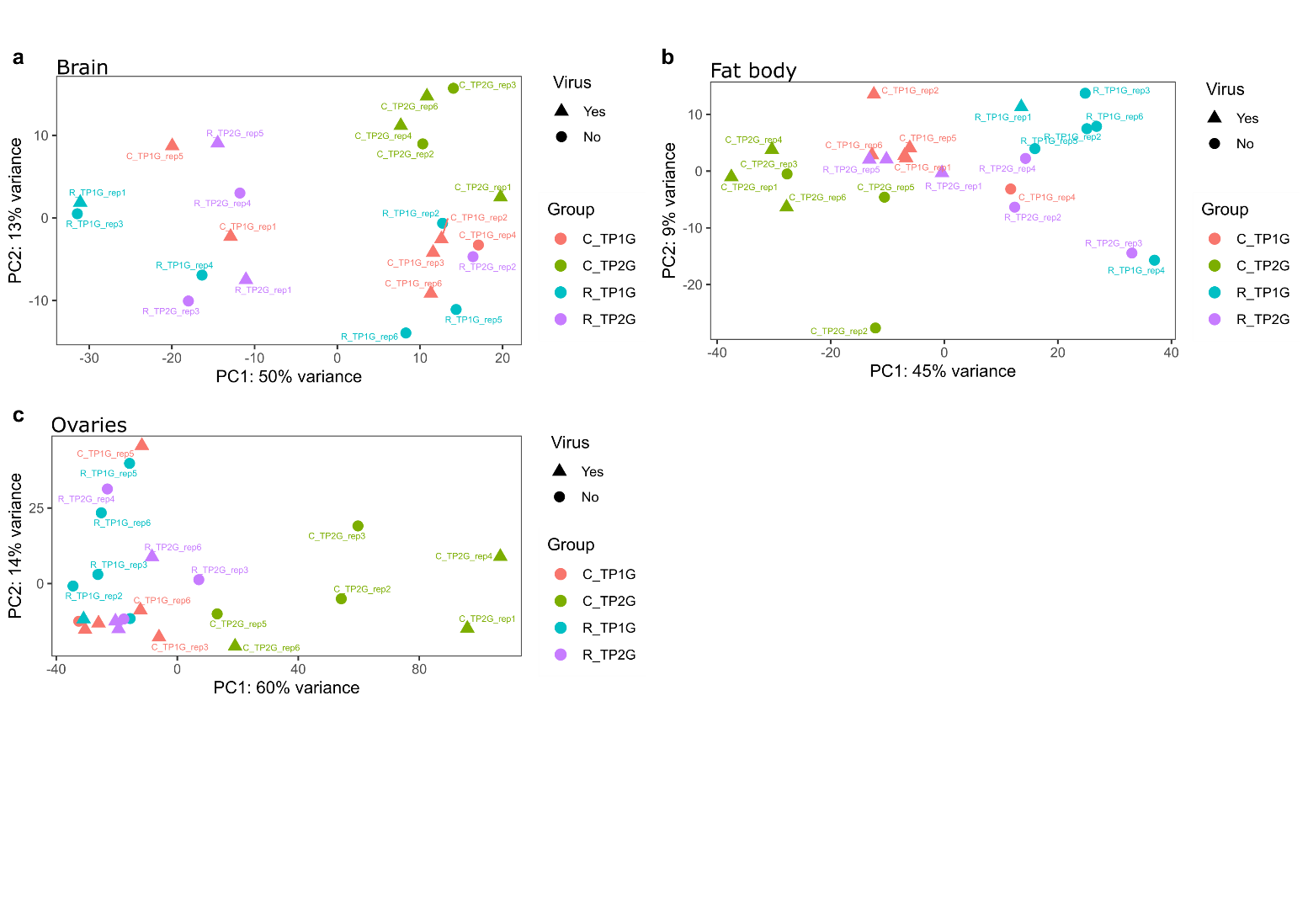


Fig. S18. Principal component analysis (PCA) for mRNA-seq libraries from single *Bombus terrestris* queens. For the mRNA-seq libraries: **a,** Brain, N = 22; **b,** Fat body, N = 24; **c,** Ovaries, N = 24. **a, b, c,** Axes represent principal components. Individual points of the same colour indicate biological replicates of the same group. Libraries where large numbers of SBPV reads were present (85,665 – 22,546,361 aligned reads) are denoted with triangles (yes; ‘with virus’ samples), whereas libraries without large numbers of SBPV reads (3 – 6,718 aligned reads) are denoted with circles (no; ‘no virus’ samples). Libraries are labelled with the library name (with, if required, a line connecting the library name and the relevant point). Library names are in the format, treatment_time-point_biological replicate. Group names in the format treatment_time-point. R, removal queens (eggs removed); C, control queens (eggs removed and replaced); TP1G, time-point 1; TP2G, time-point 2; rep1, biological replicate 1. The plots show that the mRNA-seq libraries did not cluster by presence of slow bee paralysis virus (SBPV) reads.

### Fig. S19

Fig. S19. Gene expression profile comparisons by relative age, treatment, and SBPV status in *Bombus terrestris* queens. Euler diagrams of overlaps of differentially expressed genes (DEGs) between all samples ('All Samples'), samples with very large numbers (85,665 - 22,546,361) of slow bee paralysis virus (SBPV)-aligning reads ('With virus'), and samples with very low numbers (3 – 6,718) of SBPV-aligning reads ('No virus') for three tissues. In sample names: R, removal queens (eggs removed); C, control queens (eggs removed and replaced); TP1G, time-point 1; TP2G, time-point 2. **a, b,**brain R:TP1G vs. R:TP2G (All Samples, N = 6 vs. 5; No virus, N = 5 vs. 3); **c, d,**brain R:TP2G vs. C:TP2G (All Samples, N = 5 vs. 5; No virus, N = 3 vs. 2, With virus, N = 3 vs. 3); **e, f,**fat body R:TP1G vs. R:TP2G (All Samples, N = 6 vs. 6; No virus, N = 5 vs. 3); **g, h,**fat body R:TP2G vs. C:TP2G (All Samples, N = 6 vs. 4; No virus, N = 3 vs. 3); **i, j,**ovaries R:TP1G vs. R:TP2G (All Samples, N = 6 vs. 6; No virus, N = 5 vs. 3); **k, l,**ovaries R:TP2G vs. C:TP2G (All Samples, N = 6 vs. 6; No virus, N = 3 vs. 3, With virus, N = 3 vs. 3). Up-regulated, significantly more expressed in TP2G than TP1G; down-regulated, significantly more expressed in TP1G than TP2G. Across comparisons with >50 DEGs, ‘With virus’ samples returned a mean (range) of 7.86% (2.45 - 12.7%) of DEGs not returned by All Samples (panels **c**, **d**, **k** and **l**), and ‘No virus’ samples returned a mean (range) of 3.41% (0.24 - 8.23%) of DEGs not returned by All Samples (panels **c**, **d**, **e**, **f**, **g**, **h**, **k** and **l**).

### Fig. S20


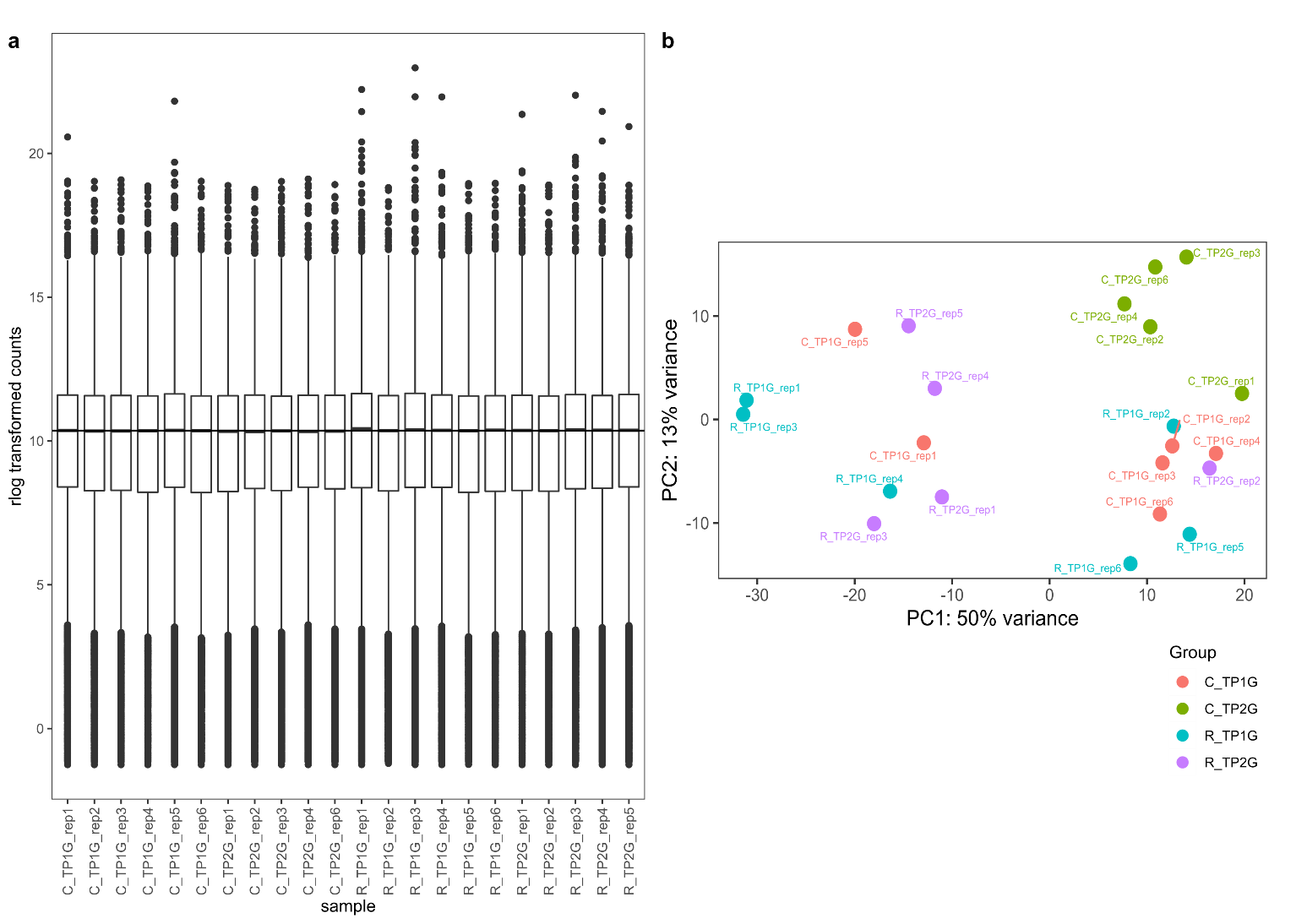


Fig. S20. Exploratory plots from the differential gene expression analysis in brain of single *Bombus terrestris* queens. **a,** Normalisation boxplots of the rlog-transformed value of mRNA-seq expression for genes in each library. Black horizontal bars: medians; boxes: interquartile ranges; whiskers: 10^th^ to 90^th^ percentile ranges. **b,** Principal component analysis (PCA) plot of the top 2,000 most highly expressed genes isolated from mRNA-seq libraries in brain. Axes represent principal components. Individual points represent biological replicates (coloured by group). Libraries are labelled with the library name (with, if required, a line connecting the library name and the relevant point). **a, b,**  Library names are in the format, treatment_time-point_biological replicate. Group names in the format treatment_time-point. R, removal queens (eggs removed); C, control queens (eggs removed and replaced); TP1G, time-point 1; TP2G, time-point 2; rep1, biological replicate 1. Brain mRNA-seq libraries: N = 22.

### Fig. S21


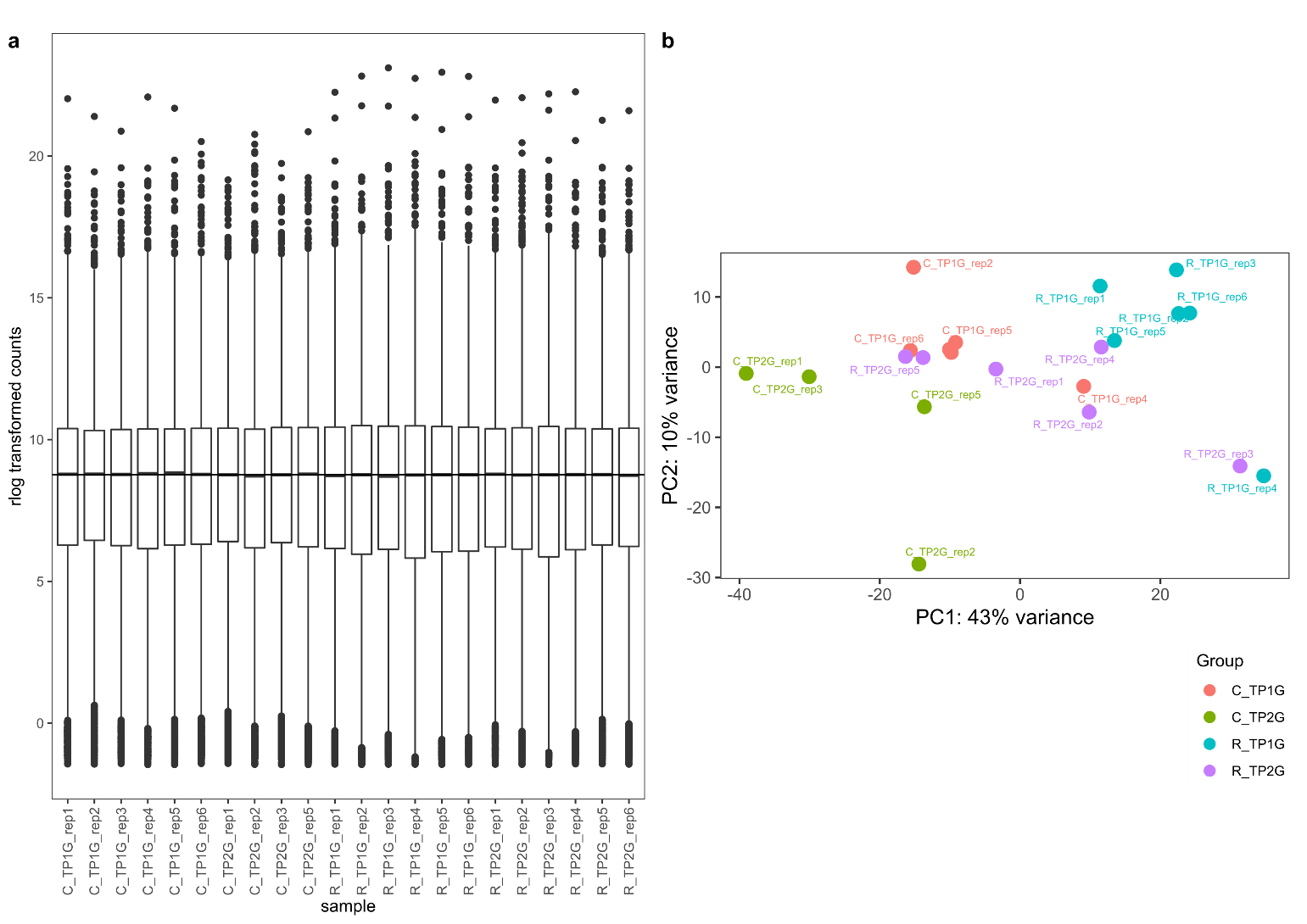


Fig. S21. Exploratory plots from the differential gene expression analysis in fat body of single *Bombus terrestris* queens. **a,** Normalisation boxplots of the rlog-transformed value of mRNA-seq expression for genes in each library. Black horizontal bars: medians; boxes: interquartile ranges; whiskers: 10^th^ to 90^th^ percentile ranges. **b,** Principal component analysis (PCA) plot of the top 2,000 most highly expressed genes isolated from mRNA-seq libraries in fat body. Axes represent principal components. Individual points represent biological replicates (coloured by group). Libraries are labelled with the library name (with, if required, a line connecting the library name and the relevant point). **a, b,**  Library names are in the format, treatment_time-point_biological replicate. Group names in the format treatment_time-point. R, removal queens (eggs removed); C, control queens (eggs removed and replaced); TP1G, time-point 1; TP2G, time-point 2; rep1, biological replicate 1. Fat body mRNA-seq libraries: N = 24.

### Fig. S22


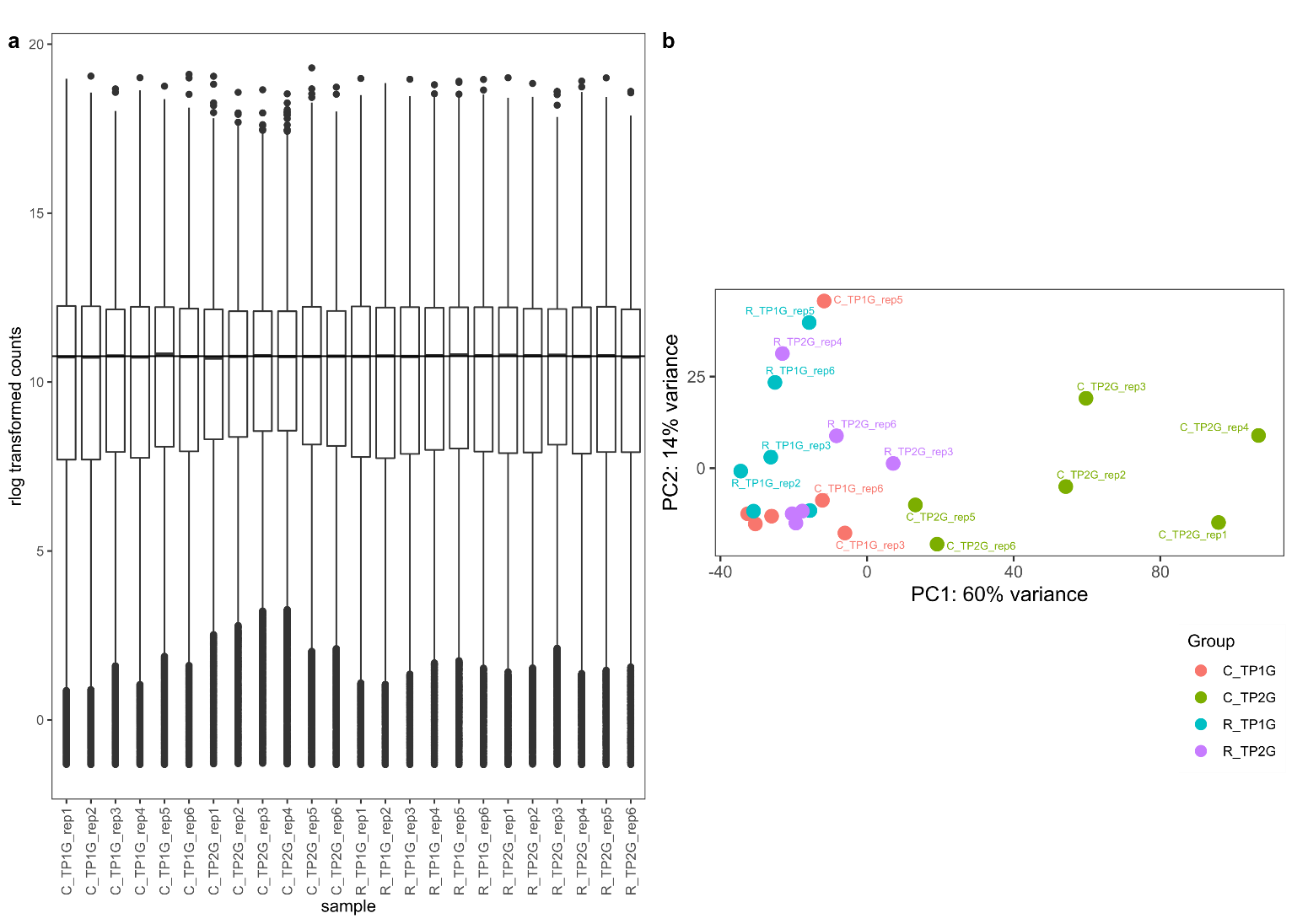


Fig. S22. Exploratory plots from the differential gene expression analysis in ovaries of single *Bombus terrestris* queens. **a,** Normalisation boxplots of the rlog-transformed value of mRNA-seq expression for genes in each library. Black horizontal bars: medians; boxes: interquartile ranges; whiskers: 10^th^ to 90^th^ percentile ranges. **b,** Principal component analysis (PCA) plot of the top 2,000 most highly expressed genes isolated from mRNA-seq libraries in ovaries. Axes represent principal components. Individual points represent biological replicates (coloured by group). Libraries are labelled with the library name (with, if required, a line connecting the library name and the relevant point). **a, b,**  Library names are in the format, treatment_time-point_biological replicate. Group names in the format treatment_time-point. R, removal queens (eggs removed); C, control queens (eggs removed and replaced); TP1G, time-point 1; TP2G, time-point 2; rep1, biological replicate 1. Ovaries mRNA-seq libraries: N = 24.
